# Should We ‘Eat a Rainbow’? An Umbrella Review of the Health Effects of Colorful Bioactive Pigments in Fruits and Vegetables

**DOI:** 10.3390/molecules27134061

**Published:** 2022-06-24

**Authors:** Michelle Blumfield, Hannah Mayr, Nienke De Vlieger, Kylie Abbott, Carlene Starck, Flavia Fayet-Moore, Skye Marshall

**Affiliations:** 1 Department of Science, Nutrition Research Australia, Sydney, NSW 2000, Australia; michelle@nraus.com (M.B.); hannah.mayr@health.qld.gov.au (H.M.); nienke.devlieger@newcastle.edu.au (N.D.V.); kylie@nraus.com (K.A.); carlene@nraus.com (C.S.); skye@nraus.com (S.M.); 2 Bond University Nutrition and Dietetics Research Group, Faculty of Health Sciences and Medicine, Bond University, Gold Coast, QLD 4226, Australia; 3 School of Clinical Medicine, University of Queensland, Brisbane, QLD 4072, Australia; 4 Centre for Functioning and Health Research, Metro South Hospital and Health Service, Buranda, QLD 4102, Australia; 5 School of Environmental and Life Sciences, University of Newcastle, Callaghan, NSW 2308, Australia; 6 Research Institute for Future Health, Gold Coast, QLD 4227, Australia

**Keywords:** fruit, vegetables, color, health, phytochemicals, carotenoids, flavonoids, chlorophyll, systematic review

## Abstract

Health promotion campaigns have advocated for individuals to ‘eat a rainbow’ of fruits and vegetables (FV). However, the literature has only focused on individual color pigments or individual health outcomes. This umbrella review synthesized the evidence on the health effects of a variety of color-associated bioactive pigments found in FV (carotenoids, flavonoids, betalains and chlorophylls), compared to placebo or low intakes. A systematic search of PubMed, EMBASE, CINAHL and CENTRAL was conducted on 20 October 2021, without date limits. Meta-analyzed outcomes were evaluated for certainty via the GRADE system. Risk of bias was assessed using the Centre for Evidence-Based Medicine critical appraisal tools. A total of 86 studies were included, 449 meta-analyzed health outcomes, and data from over 37 million participants were identified. A total of 42% of health outcomes were improved by color-associated pigments (91% GRADE rating very low to low). Unique health effects were identified: *n* = 6 red, *n* = 10 orange, *n* = 3 yellow, *n* = 6 pale yellow, *n* = 3 white, *n* = 8 purple/blue and *n* = 1 green. Health outcomes associated with multiple color pigments were body weight, lipid profile, inflammation, cardiovascular disease, mortality, type 2 diabetes and cancer. Findings show that color-associated FV variety may confer additional benefits to population health beyond total FV intake.

## 1. Introduction

Inadequate intake of fruits and vegetables (FV) is a leading modifiable dietary risk factor for mortality and contributes to the increasing burden of both communicable and non-communicable diseases [1,2]. In 2017, poor FV intake was responsible for 3.9 million deaths [3] and was among the top dietary risk factors affecting disability-adjusted life years worldwide [1]. Not only is meeting recommended servings of FV important, but a greater variety in the types of FV consumed has been independently associated with a lower risk of diabetes [4], cancer [5,6,7] and mortality [8,9], and improved cognitive function [10,11]. Increasing variety of FV is particularly critical during childhood to support growth and development, and to establish healthy eating habits that track into adulthood [12].

FV contain an abundance of nutrients, including vitamins, minerals and bioactive compounds known as phytonutrients. Phytonutrients improve health through their antioxidant, anti-inflammatory, antibacterial, antifungal, antiallergic, chemoprotective, neuroprotective, hypolipidemic and/or hypotensive properties [13]. Despite the unequivocal health benefits of eating FV, 78% of adults worldwide do not consume the daily recommended servings [14], leading to a ‘phytonutrient gap’. Naturally occurring and pigmented phytonutrients (herein referred to as bioactive pigments) give FV their vibrant colors and correspond to one or more phytonutrient categories; e.g., red corresponds to lycopene, yellow to alpha-carotene, orange to beta-carotene, green to chlorophyll, purple and blue to anthocyanins, and white to flavones (Table 1) [15]. Given the range of colors and bioactive pigments in FV, both the nutrient profile and physiological functions of FV may differ in part due to their variations in color, and those of the same color are likely to have similar health benefits.

Population-based data have shown that the diets of eight out of ten American adults fall short in every color of phytonutrient (i.e., have a phytonutrient gap), when compared to the median phytonutrient intake in adults who meet the recommended daily intake of FV, with the proportion of insufficient intakes per color category reported as 88% for the color purple/blue, 86% for white, 79% for yellow/orange, 78% for red and 69% for green [17]. In an attempt to improve health, dietary guidelines and health promotion campaigns have advocated for individuals to ‘eat by color’ or ‘eat a rainbow’ of FV [15]. Associating each color with a health benefit is a simplified strategy designed to: (1) help individuals relate to the health properties of FV, (2) promote greater recognition of their importance, and (3) increase the diversity of FV colors consumed across all life stages [15]. Despite these campaigns, assessment of FV variety in both clinical practice and research has been typically based on the number of individual types of FV a person consumes rather than assessing variety of bioactive pigments [18,19]. Observational studies have shown that FV intakes, grouped by their color, are associated with improvement in a range of health outcomes including cognitive decline, cardiovascular disease (CVD) and colorectal cancer [20,21,22,23]. The body of evidence linking bioactive pigments in FV to beneficial health effects is growing, but the reviews and syntheses of the evidence have focused either on individual pigments or on individual health outcomes [24,25,26,27,28,29,30,31,32]. There is a gap in practice and in research whereby the evidence for consuming a variety of color and bioactive pigments from FV for human health and wellbeing is summarized and synthesized. 

Collating the evidence will support recommendations for improving health related to the types of bioactive pigments found in FV and highlight important research opportunities. Findings for each bioactive pigment color are relevant to all FV which contain them, and are not limited to a specific FV, thereby increasing the translational impact of existing messaging around eating a variety of FV. The aim of this umbrella review was to synthesize the evidence on the effects of a variety of color-associated bioactive pigments found in FV (carotenoids, flavonoids, betalains and chlorophylls), as compared to placebo or low intakes, on human health outcomes relevant to population health.

## 2. Results

**Figure 1:**
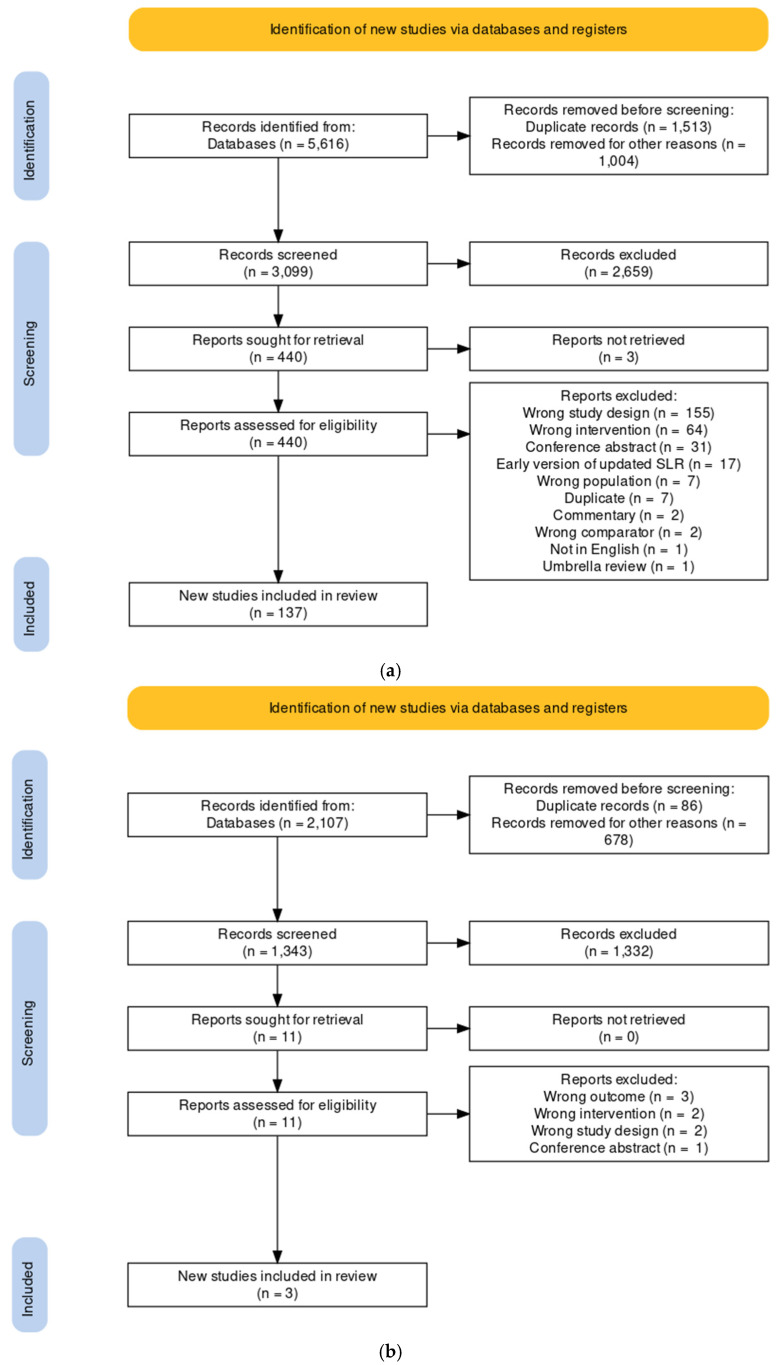
PRISMA flow diagram of the literature search and selection. (**a**) Flow chart for the search for systematic literature reviews with meta-analyses for all color pigments. (**b**) Flow chart for the search for randomized controlled trials and cohort studies for the green pigment chlorophyll.

The systematic search strategy identified 5616 records, of which 137 systematic literature reviews (SLRs) containing 449 meta-analyses (MAs) were eligible for inclusion (Figure 1a). Fifty-four SLRs were excluded due to a very high degree of overlapping of studies included in MAs of the same pigment and health outcome, resulting in 83 SLRs included in this umbrella review. When the search was extended to include single randomized controlled trials (RCTs) or cohort studies for chlorophyll, three additional studies were included: two RCTs and one cohort (Figure 1b).

### 2.1. Characteristics of Included Studies

Characteristics of included studies are presented in the data extraction spreadsheet published elsewhere [33]. The included SLRs were published between 1998 and 2021 and were conducted in adults. The number of primary studies within the included SLRs ranged from 2 to 38. Of the 83 included SLRs, *n* = 33 included only RCTs, *n* = 12 only cohort studies, *n* = 6 only case–control studies and *n* = 32 both cohort and case–control studies. The number of SLRs and MAs included in this umbrella review, by pigment and health outcome are summarized in Table S1. 

MAs included participants of both sexes, except for outcomes relating to pregnancy health (females only), breast or ovarian cancer (females only), prostate cancer (males only) and a single MA of osteoporosis (males only [34]). None of the included MAs reported on health effects in children or adolescents, and only *n* = 7 MAs (0.02%) were reported exclusively in older adults. The countries of the original research were rarely and poorly reported by the included SLRs, and therefore were not extracted.

For chlorophyll, included RCT (*n* = 2) and cohort (*n* = 1) studies were published between 2006 and 2016, and were conducted in adults of both sexes from the Netherlands, Sweden and Japan.

### 2.2. Bioactive Pigment Interventions

#### 2.2.1. SLRs and MAs

The review of SLRs identified MAs on 17 different bioactive pigments which included all colors of fruits and vegetables except green (i.e., chlorophyll) (Table S1). No MAs were found for betalains or it’s sub-classes; however, the betalains colors of red, violet, orange, and yellow were represented by the included carotenoids and flavonoids. 

The only major class of bioactive pigment included for data extraction was carotenoids (*n* = 4 SLRs reporting *n* = 12 MAs), as all other health outcomes identified were reported at the bioactive pigment subclass. The bioactive pigment sub-classes which reported unique health outcomes were flavonols, and they were all pale yellow in color (kaempferol *n* = 1 SLR reporting *n* = 1 MA, myricetin *n* = 1 SLR reporting *n* = 1 MA, quercetin *n* = 10 SLRs reporting *n* = 25 MAs) and tannins (proanthocyanidins *n* = 5 SLRs reporting *n* = 11 MAs, proanthocyanins *n* = 2 SLRs reporting *n* = 2 MAs), which can be red, blue, purple or brown (Table S1).

Anthocyanin (red/blue/purple) was the most extensively researched bioactive pigment (*n* = 18 SLRs reporting *n* = 81 MAs, representing *n* = 729 original research studies), followed by beta-carotene (orange; *n* = 34 SLRs reporting *n* = 74 MAs) and lycopene (red; *n* = 25 SLRs reporting *n* = 65 MAs). Of the bioactive pigment subclasses included, zeaxanthin (yellow; *n* = 2 SLRs reporting *n* = 3 MAs) was the least explored (Table S1); and no MAs were identified for the sub-classes capsorubin, capsanthin, violaxanthin, aurones and chalcones.

Bioactive pigments were primarily investigated via dietary intake (*n* = 20 MAs of RCTs, *n* = 186 MAs of cohort studies), followed by natural supplements or a mix of natural and synthetic supplements (*n* = 74 MAs of RCTs, *n* = 1 MA of observational research), and serum levels (*n* = 47 MAs of observational studies). However, a large number of MAs included bioactive pigments measured from a variety of sources including diet, supplement and/or serum levels (*n* = 68 MAs of observational research, *n* = 53 MAs of RCTs). 

Intervention duration varied widely, from 4 h to 18 years in RCTs, and from 3 months to 41 years in observational studies. Comparator groups were either placebo or non-specified controls for RCTs, and the lowest category of intake for cohort studies.

#### 2.2.2. Single RCTs and Cohort Studies 

Two RCTs delivered chlorophyll as a supplement containing 3000 mg extracted from green spinach leaves; and another as 0.7 mg of chlorophyll c2. A cohort study examined chlorophyll intake from the usual diet.

### 2.3. Health Outcomes and Confidence in the Body of Evidence

#### 2.3.1. SLRs and MAs

This umbrella review of SLRs identified many unique meta-analyzed outcomes (*n* = 98), which were grouped as cancer (*n* = 192 MAs), CVD (*n* = 135 MAs), exercise (*n* = 28 MAs), mortality (*n* = 27 MAs), type 2 diabetes mellitus (T2DM; *n* = 24 MAs), obesity (*n* = 13 MAs), bone health (*n* = 9 MAs), eye health (*n* = 9 MAs), the nervous system (*n* = 5 MAs), pregnancy health (*n* = 4 MAs), cognitive function (*n* = 2 MAs) and the respiratory system (*n* = 1 MA) (Table S1). 

Of the 449 MAs included, 42% (*n* = 89 MAs) reported at least one significant improvement in a pooled health outcome, with *n* = 35 (19%) having a significant dose–response. There was also *n* = 4 MAs (0.009% of included MAs) which reported a significant negative effect from having the bioactive pigment (Tables S2–S8).

Using GRADE, confidence in the body of evidence ranged from very low (*n* = 349 MAs), low (*n* = 61 MAs), medium (*n* = 28 MAs), and high (*n* = 11 MAs). Of the 28 included SLRs that reported their own GRADE level, the current investigators allocated a higher GRADE rating for seven MAs, agreed with the GRADE rating for six MAs, and allocated a lower GRADE rating for 15 MAs. The most common reason for downgrading the confidence in the body evidence was that most (67%) MAs were based on observational data, which downgraded all GRADE ratings to at least “low” confidence. Other common reasons for downgrading were moderate to high risk of bias in the original studies included in the SLRs, wide confidence intervals, or substantial statistical heterogeneity.

#### 2.3.2. Single RCTs and COHORT STUDIES

Each included RCT and cohort study for chlorophyll examined a unique health outcome: cancer (*n* = 1 case–cohort study), CVD (*n* = 1 RCT) and allergy (*n* = 1 RCT) (Table S9). Of the 16 included health variables extracted from these three studies, only one was significant, with a second variable with borderline significance and likely underpowered by a small sample size (*n* = 36 participants; *p* = 0.06). As no two included original research studies on chlorophyll examined the same health outcome, meta-analysis and GRADE assessment were not performed.

### 2.4. Health Effects of Total Carotenoid Pigments in Fruits and Vegetables

**Figure 2:**
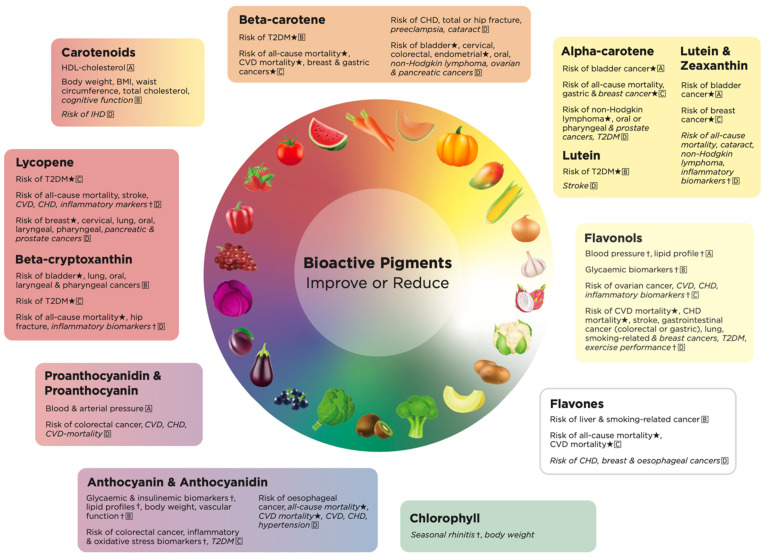
The health promoting effects of bioactive pigments by color in fruits and vegetables. GRADE working groups of evidence: A = high quality, further research is unlikely to change our confidence in the estimated effect; B = medium quality, further research is likely to have an important impact on our confidence in the estimate of effect and may change the estimate; C = low quality, further research is very likely to have an important impact on our confidence in the estimate of effect and is likely to change the estimate; and D = very low quality, we are very uncertain about the estimate. ⋆ = dose–response established. † = cause and effect established. Health effects in italics are those with small effect sizes. BMI, body mass index. CVD, cardiovascular disease. CHD, coronary heart disease. IHD, ischemic heart disease. T2DM, type 2 diabetes.

Total carotenoids represent red, orange, and yellow pigments (Table 1, Figure 2). There was *n* = 12 unique MAs (*n* = 10 MAs of RCTs, *n* = 2 MAs of cohort studies) which were reported by the bioactive pigment class carotenoids. Carotenoid intervention was measured via dietary intake (*n* = 1 MA with 4–24 years follow-up), serum (*n* = 1 MA with 12–14 years follow-up), supplement (*n* = 8 MAs with 12–16 weeks intervention duration) or mixed (*n* = 2 MAs with 2-months to 18-years intervention duration) (Tables S1 and S2). The intervention doses in RCTs were not reported (*n* = 5 MAs) or were 0.5 mg to 60 mg/day, and cohort studies compared the highest categories of intake or serum levels with the lowest.

Carotenoid supplementation had a large effect on risk factors for obesity and CVD, including reductions in body weight (SMD −2.34 kg; 95% CI −3.80, −0.87), BMI (SMD −0.95 kg/m^2^; 95% CI −1.88, −0.01), waist circumference (SMD −1.84 cm; 95% CI −3.14, −0.54), total cholesterol (SMD −2.10 mg/dL; 95% CI −3.20, −0.99), and increased HDL cholesterol (SMD 0.76 mg/dL; 95% CI 0.10, 1.41) when consumed for up to 16 weeks [35] (Table S2).

The highest category of dietary carotenoid intake was associated with a 15% decreased risk of ischemic heart disease (RR 0.85; 95% CI 0.77, 0.93), compared to the lowest category of intake [36]. High carotenoid levels (0.5–50 mg) modestly improved cognitive outcomes (SMD 0.14, 95% CI, 0.08, 0.20) [28] (Table S2). Total carotenoid intake was found to have no effect on tumor necrosis factor alpha (TNF-alpha), triglycerides, low density lipoprotein (LDL) cholesterol or change in fat ratio [35,37] (Table S2). No dose–response MAs were included for total carotenoids.

The strongest evidence for the health effect of carotenoids was for improved adiposity (very large effect sizes, GRADE: low to medium) and lipid profiles (large to very large effect sizes, GRADE: medium to high) (Figure 2).

### 2.5. Health Effects of Red Pigments in Fruits and Vegetables

Data on the effect of red bioactive pigments were from beta-cryptoxanthin (*n* = 15 SLRs reporting *n* = 33 MAs) and lycopene (*n* = 25 SLRs of *n* = 65 MAs) (Tables S1 and S3). Anthocyanins may also be red in an acidic environment, but were reported with the blue/purple bioactive pigments [38].

#### 2.5.1. Beta-Cryptoxanthin

Only two of the included MAs on beta-cryptoxanthin were based on RCT data (12-weeks; 6 mg/day; mixed sources of beta-cryptoxanthin), and the remaining 31 MAs were based on cohort data (1–26 years). Most cohort MAs compared an unspecified highest category with the lowest; however, where the highest categories were specified, they provided 56–200 µg/day compared with the lowest at <1.8 to 20 µg/day. Cohort data were derived from diet (*n* = 21 MAs), mixed sources (*n* = 3 MAs), or serum levels (*n* = 7 MAs); and included seven dose–response MAs.

The highest category of beta-cryptoxanthin intake was associated with a 28% decreased risk of hip fracture (OR 0.72; 95% CI 0.60, 0.87) [39] and up to a 27% decreased risk of all-cause mortality (RR 0.73; 95% CI, 0.58, 0.88) [40], compared to the lowest category of intake. A small effect was also found for the inflammatory biomarker C-reactive protein (CRP; MD −0.35 mg/L; 95% CI −0.54, −0.15), after individuals consumed 6 mg beta-cryptoxanthin over 12-weeks [37] (Table S3). 

In relation to cancer, the highest category of dietary beta-cryptoxanthin intake was associated with a 69% decreased risk of larynx cancer (OR 0.41; 95% CI 0.33, 0.51) [41], 64% decreased risk of oral cavity and pharynx cancer (OR 0.46; 95% CI 0.29, 0.74) [41], 42% decreased risk of bladder cancer (RR 0.58; 95% CI 0.36, 0.94) [42], and 20% decreased risk of lung cancer (RR 0.80; 95% CI 0.72, 0.89) [43], compared to the lowest category of intake (Table S3).

In dose–response MAs of serum levels, each 0.1 mg/day increase in beta-cryptoxanthin, decreased the risk of all-cause mortality by 6% (RR 0.94; 95% CI 0.89, 0.99) [40], whereas for every daily increase of 0.5 µmol/L, the risk of T2DM decreased by 15% (RR 0.85; 95% CI 0.76, 0.94) [44] (Table S3).

No differences were found for beta-cryptoxanthin and risk of: cataracts [45], early age-related macular degeneration [46], osteoporosis [39], Parkinson’s disease [47], non-Hodgkin lymphoma [48], breast cancer [29], colorectal cancer [49], pancreatic cancer [50] or lung cancer mortality [43] (Table S3). 

The strongest evidence for the health effect of beta-cryptoxanthin was for a decreased risk of all-cause mortality (dose–response relationship, moderate to large effect size, GRADE: very low), bladder cancer (dose–response relationship, very large effect size, GRADE: medium), oral, laryngeal, or pharyngeal cancer (very large effect size, GRADE: very low to medium), and T2DM (dose–response relationship, large effect size, GRADE: low) (Figure 2).

#### 2.5.2. Lycopene

There was *n* = 14 MAs included based on RCT data (1-day to 6-months duration; 2–50 mg/day); with the remaining *n* = 51 MAs based on observational cohort data (3-months to 26-years duration; 2035–10,000 µg/day), *n* = 11 of which were dose–response MAs (per 1000 µg/day or incremental serum levels) (Table S3). Most MAs analyzed dietary intake data (*n* = 32 MAs), followed by mixed sources (*n* = 22 MAs), serum values (*n* = 10 MAs), and one MA measured supplemental intake.

The highest category of dietary lycopene intake was associated with reductions in the risk of cervical (OR 0.54; 95% CI, 0.39, 0.75) [51], larynx (OR 0.50; 95% CI, 0.28, 0.89) [41], lung (RR 0.71; 95% CI, 0.51, 0.98) [43], oral cavity and pharynx (OR 0.74; 95% CI, 0.56, 0.98) [41] and prostate (RR 0.88; 95% CI, 0.79, 0.99) [34] cancers, compared to the lowest category of dietary lycopene intake. Reductions in the risk of breast cancer were only reported in case control studies, where greater reductions in risk up to 29% (OR 0.71; 95% CI, 0.56, 0.92) [29] were found with greater dietary lycopene intake (Table S3). 

Higher lycopene intake was also associated with cardiovascular improvements with small to moderate clinical significance, including a lower risk of CHD (RR 0.87; 95% CI, 0.76, 0.98) [52], CVD (HR 0.86; 95% CI 0.77, 0.95) [53], stroke (HR 0.74; 95% CI 0.62, 0.89) [53], T2DM (RR 0.85; 95% CI 0.76, 0.96) [44], mortality (HR 0.63; 95% CI 0.49, 0.81) [53] and all-cause mortality (RR 0.72; 95% CI 0.49, 0.95) [40] (Table S3). In dose–response MAs of serum levels, each 0.5 µmol/L increase in serum lycopene decreased the risk of T2DM by 17% (RR 0.83; 95% CI 0.74, 0.92) [44].

Lycopene status had no effect on preeclampsia [54], early age-related macular degeneration [46], risk of hip fracture [55], advanced prostate cancer [34,56], colon/colorectal/rectal cancer [49], bladder cancer [42], gastric cancer [57], non-Hodgkin lymphoma [48], ovarian cancer [58], Parkinson’s disease [47], inflammatory biomarkers (except a small effect in interleukin-6 (MD −1.08 pg/mL; 95% CI −2.03, −0.12) [37], lipid profiles [59,60], blood pressure [60] and prostate specific antigen (PSA) levels [61] (Table S3).

The strongest evidence for the health effects of lycopene were decreased risk of breast cancer (dose–response relationship, large to very large effect size, GRADE: very low) and T2DM (dose–response relationship, moderate to large effect size, GRADE: very low to low) (Figure 2).

### 2.6. Health Effects of Orange Pigments in Fruits and Vegetables

The health effects of consuming orange bioactive pigments from FV were reported by MAs of beta-carotene (bioactive pigment subclass; *n* = 34 SLRs reporting *n* = 75 MAs including *n* = 16 dose–response MAs) (Tables S1 and S4).

Evidence for the effects of beta-carotene was largely represented by MAs of cohort studies (*n* = 59 MAs) measured over 1–26 years via dietary intake (*n* = 32 MAs), mixed sources (*n* = 16 MAs), serum levels (*n* = 10 MAs) or supplementation (*n* = 1 MA). Doses in the highest categories of intake were not usually reported, but when reported ranged from 2473–7000 µg/day intake or 16 to >120 µg/dL serum level. The one supplemental study provided a dose of 600 to 1991 µg/day (Table S4). Sixteen of the observational MAs were dose–response, examining effects per 500–5000 µg/day intake or per 0.1 µmol/L serum level.

The highest category of beta-carotene intake was associated with a decreased risk of several types of cancers, including cervical (OR 0.68; 95% CI 0.55, 0.84) [51], gastric (OR 0.74; 95% CI 0.61, 0.91) [62], larynx (OR 0.43; 95% CI 0.24, 0.77) [41], non-Hodgkin lymphoma (RR 0.80; 95% CI 0.68, 0.94) [48], oral cavity (OR 0.54; 95% CI 0.37, 0.80) [41], ovarian (RR 0.84; 95% CI 0.75, 0.94) [63] and pancreatic (OR 0.78; 95% CI 0.66, 0.92) [50] cancers, compared to the lowest category of beta-carotene intake (Table S4). Reductions in breast cancer risk were supported by dose–response MAs that found dietary beta-carotene intakes of 2000 µg/day, 3000 µg/day or 5000 µg/day reduced the risk of breast cancer by 3%, 4% and 7%, respectively [29] (Table S4). For each 1000 µg/1000 kcal increase in dietary beta-carotene the risk of endometrial cancer decreased by 26% (RR 0.74; 95% CI 0.61, 0.91) [64].

Highest categories of beta-carotene intake were also associated with a lower risk of all-cause mortality (RR 0.82; 95% CI 0.77, 0.86) [40], CHD (RR 0.73; 95% CI 0.65, 0.82) [65], CVD mortality (RR 0.68; 95% CI 0.52, 0.83) [66], total fracture (RR 0.63; 95% CI 0.52, 0.77) [67], hip fracture (OR 0.72; 95% CI 0.54, 0.95) [55] and the incidence of cataract (RR 0.90; 95% CI 0.83, 0.99) [45] and preeclampsia (SMD −0.40; 95% CI −0.72, −0.08) [54], when compared to the lowest intakes. In dose–response MAs, each 1 mg/day increase in beta-carotene intake decreased the risk of all-cause mortality by 5% (OR 0.95; 95% CI 0.92, 0.99) [40], whereas for every 0.5 µmol/L serum increase the risk of T2DM decreased by 35% (OR 0.65; 95% CI 0.48, 0.89) [44] (Table S4).

No differences were found for dietary beta-carotene and risk of bladder cancer [42], colon cancer [49], colorectal cancer [49], lung cancer [43], lung cancer or lung cancer mortality [43], melanoma [68], prostate cancer [56,62], rectal cancer [49], COPD [69], total fracture [70] and Alzheimer’s disease [71] (Table S4).

The strongest evidence for the health effects of beta-carotene were decreased risk of all-cause and CVD mortality (dose–response relationship, very large effect size, GRADE: very low to low), T2DM (dose–response relationship, large to very large effect size, GRADE: low to medium), bladder cancer (dose–response relationship, very large effect size, GRADE: very low), breast cancer (dose–response relationship, large effect size, GRADE: very low to low) and endometrial cancer (dose–response relationship, large effect size, GRADE: very low) (Figure 2).

### 2.7. Health Effects of Yellow Bioactive Pigments in Fruits and Vegetables

The evidence for the health effects of yellow bioactive pigments were from MAs reporting on alpha-carotene (*n* = 16 SLRs reporting *n* = 41 MAs), lutein (*n* = 7 SLRs reporting *n* = 10 MAs), zeaxanthin (*n* = 2 SLRs reporting *n* = 3 MAs) or lutein and zeaxanthin as a combined group (*n* = 13 SLRs reporting *n* = 31 MAs) (Figure 2; Table S5). 

#### 2.7.1. Alpha-Carotene

All *n* = 41 MAs reporting on the health effects of alpha-carotene were based on cohort and/or case–control research, measured via dietary intake (*n* = 27 MAs with 1–25 years follow-up); serum levels (*n* = 10 MAs with 2–26 years follow-up); or mixed diet, serum levels, and/or supplements (*n* = 4 MAs with 9-months to 26-years follow-up) (Table S5). Most MAs compared an unspecified highest category against the unspecified lowest category; however, some category groups were defined as >881–2000 µg/day compared against <180 to 300 µg/day dietary intake or serum levels of >1 to >5 µg/dL compared against <1 to <2 µg/dL. 

The highest category of alpha-carotene intake was associated with a reduced risk of gastric (OR 0.58; 95% CI 0.44, 0.76) [57], non-Hodgkin lymphoma (RR 0.87; 95% CI 0.78, 0.97) [48], oral cavity and pharynx (OR 0.57; 95% CI 0.41, 0.79) [41] and prostate (RR 0.87; 95% CI 0.76, 0.99) [56] cancers, and a reduced risk of T2DM (RR 0.91; 95% CI 0.85, 0.96) [44] and all-cause mortality (RR 0.79; 95% CI, 0.63, 0.94) [40]. In dose–response MAs, for each 1000 µg/day increase in alpha-carotene the risk of breast cancer decreased by up to 18% (RR 0.82; 95% CI 0.73, 0.93) [29] and the risk of non-Hodgkin lymphoma decreased by 13% (RR 0.87; 95% CI 0.78, 0.97) [48] (Table S5).

Alpha-carotene intake was reported to have no effect on risk of pre-eclampsia [54], cataract [45], early aged-related macular degeneration [46], cancer of the larynx [41], risk of colon, rectal, or colorectal cancer [49], hip fracture [55], lung cancer [43], pancreatic cancer [50] or Parkinson’s disease [47].

The strongest evidence for the health effects of alpha-carotene were decreased risk of all-cause mortality (dose–response relationship, large effect size, GRADE: very low to low), bladder cancer (dose–response relationship, very large effect size, GRADE: high), non-Hodgkin lymphoma (dose–response relationship, moderate to large effect size, GRADE: very low) and T2DM (dose–response relationship, large to very large effect size, GRADE: medium) (Figure 2).

#### 2.7.2. Lutein

All *n* = 10 MAs reporting on the health effects of lutein drew upon cohort or case–control data, measured via dietary intake (*n* = 4 MAs with 10–12 years follow-up), serum levels (*n* = 2 MAs with 8–10 years follow-up) or mixed sources (*n* = 4 MAs with 9-months to 26-years follow-up) (Table S5). Most of the MAs compared the highest unspecified category of lutein to the lowest unspecified category; however, one highest category was defined as 3701 to 4041 µg/day compared to 1413 to 1736 µg/day dietary intake.

When compared to the lowest category, the highest category of lutein intake improved the risk of stroke by 18% (RR 0.82; 95% CI 0.58, 0.78) [72] and the risk of T2DM by 35% (RR 0.65; 95% CI 0.55, 0.77). In dose–response MAs of serum levels, each 0.2 ugmol/L increase in lutein decreased the risk of T2DM by 21% (RR 0.79; 95% CI 0.72, 0.86). Lutein was reported to have no effect on risk of lung cancer [43], gastric cancer [57], Parkinson’s disease [47], pre-eclampsia [54] or all-cause mortality [40] (Table S5).

The strongest evidence for the health effects of lutein was for decreased risk of T2DM (dose–response relationship, large to very large effect size, GRADE: medium) (Figure 2).

#### 2.7.3. Zeaxanthin

All three MAs which measured the effect of zeaxanthin on human health were based on cohort studies, with *n* = 2 MAs based on serum zeaxanthin levels (8–10 years follow-up) and *n* = 1 MA based on mixed sources (2–26 years follow-up). When comparing the highest unspecified category against the lowest unspecified category, zeaxanthin had no effect on all-cause mortality [40] or risk of T2DM [44] (Table S5).

#### 2.7.4. Lutein and Zeaxanthin

Thirty-one MAs investigated the effect of combined lutein and zeaxanthin on human health, *n* = 29 of which were based on cohort of case–control studies, and *n* = 2 were based on RCTs. The observational research primarily measured lutein and zeaxanthin via dietary intake (*n* = 22 MAs with 1–25 years follow-up) or serum levels (*n* = 5 MAs with 2–26 years follow-up), with only one MA considering mixed sources (5–18 years follow-up) (Table S5). Only *n* = 3 MAs defined the intake of lutein and zeaxanthin in the highest category (>1815 to 5000 µg/day) as compared to the lowest category (<775 to 1000 µg/day). The two RCT MAs both considered dietary or supplemental intake of lutein and zeaxanthin with interventions ranging from 8–32 weeks and doses of 8 mg/day to 27 mg/day. 

When comparing the highest category versus lowest category of lutein and zeaxanthin status, higher dietary intakes reduced the risk of non-Hodgkin lymphoma by 18% (RR 0.82; 95% CI 0.69, 0.97) [48], while higher serum intakes reduced the risk of bladder cancer (RR 0.53; 95% CI 0.33, 0.84) [42] and all-cause mortality (RR 0.85; 95% CI 0.74, 0.97) [40]. Lutein and zeaxanthin intakes were associated with decreased CRP levels (SMD −0.3 mg/L; 95% CI −0.45, −0.15) [37] and a dose–response relationship found a favorable 17% decreased risk of breast cancer for every 3000 µg/day increase in lutein and zeaxanthin intake (RR 0.83; 95% CI 0.77, 0.89) [29]. Dose–response relationships were not found for any other level of lutein or zeaxanthin intake (Table S5). 

For lutein and zeaxanthin, no differences were found for the risk of gastric cancer [57], lung cancer [43], lung cancer or lung cancer mortality [43], pancreatic cancer [50], oral cavity and pharynx cancer [41], colon [49], rectal [49] or colorectal cancer [49], early aged macular degeneration [46], hip fracture [55] or IL-6 [37] (Table S5).

The strongest evidence for combined lutein and zeaxanthin was for decreased risk of bladder cancer (dose–response relationship, large to very large effect size, GRADE: low to high) and breast cancer (dose–response relationship, large effect size, GRADE: very low to low) (Figure 2).

### 2.8. Health Effects of Pale-Yellow Bioactive Pigments in Fruits and Vegetables

The health effects of consuming pale yellow bioactive pigments from FV were reported by MA of flavonols (bioactive pigment subclass; *n* = 17 SLRs reporting *n* = 33 MAs), kaempferol (*n* = 1 SLR reporting *n* = 1 MA), myricetin (*n* = 1 SLR reporting *n* = 2 MA), and quercetin (*n* = 10 SLRs reporting *n* = 25 MAs) (Tables S1 and S6).

#### 2.8.1. Flavonols

As a group, MAs of flavonols subclass were primarily based on cohort and/or case–control data (*n* = 25 MAs with 2–28 years follow-up); although there was substantial cause-and-effect investigation via *n* = 8 MAs of RCTs (14–84 days intervention duration). Over half (*n* = 14 of 25) of the observational MAs measured flavonols from dietary sources alone (1–27 years follow-up), with the remaining *n* = 11 MAs measuring flavonols from mixed sources (4–28 years follow-up) (Table S6). The doses of intake in the highest or lowest categories were not reported. Four of the observational MAs were dose–response, examining effects per 10 mg or 20 mg, and were based on cohort data. All the MAs based on RCTs tested the effect of flavonols delivered via supplementation from 14- to 90-days at doses of 6–1000 mg (Table S6).

When comparing the highest intake or levels with the lowest, flavonols were found to improve the risk of stroke (RR 0.86; 95% CI 0.75, 0.96), CVD (RR 0.85; 95% CI 0.79, 0.91), and CHD (RR 0.88; 95% CI, 0.79, 0.98), as well as CVD- (RR 0.79; 95% CI 0.63, 0.99) and CHD-related death (RR 0.80; 95% CI 0.69, 0.93) [73,74,75,76,77]. High categories of flavonols were also associated with a reduced risk of T2DM (RR 0.92; 95% CI 0.85, 0.98) [78] and risk of breast (RR 0.88; 95% CI 0.80, 0.96), colorectal (RR 0.71; 95% CI 0.63, 0.81), gastric (OR 0.80; 95% CI 0.70, 0.91), ovarian (RR 0.68; 95% CI 0.58, 0.80) and smoking related cancer (OR 0.77; 95% CI 0.63, 0.95) [79,80,81,82,83]. However, two other MAs found no association with breast cancer [84], one found no association with CHD [85], and no differences were found for effect on all-cause mortality [73], hypertension [86] or other types of cancer including liver, lung, pancreatic, esophageal or prostate [84,87,88] (Table S6).

In dose–response MAs, for each 20 mg/day increase in flavonols the risk of stroke decreased by 14% (RR 0.86; 95% CI 0.77, 0.96) [77], and for each 10 mg/day increase in flavonols the risk of CVD mortality decreased by 13% (RR 0.87; 95% CI 0.76, 0.99) [73]. MAs of RCTs examined chronic disease indicators, finding supplementation with flavonols improved systolic (MD −3.05 mmHg; 95% CI −4.83, −1.27) and diastolic (MD −2.63 mmHg; 95% CI −3.83, −1.42) blood pressure, HDL cholesterol (MD 0.05 mmol/L; 95% CI 0.02, 0.07), LDL cholesterol (MD −0.14 mmol/L; 95% CI −0.21, −0.07), and total cholesterol (MD −0.11 mmol/L; 95% CI −0.20, −0.02), blood glucose (MD −0.18 mmol/L; 95% CI −0.29, −0.08) and triglycerides (MD −0.11 mmol/L; 95% CI −0.18, −0.03) [89]; however, there was no effect on waist circumference [90] (Table S6).

#### 2.8.2. Kaempferol, Quercetin and Myricetin

One SLR reported on highest versus lowest dietary intake of kaempferol, quercetin, and myricetin using case–control data (duration not reported) [84]; whereas the nine other SLRs reported on 30–1000 mg/day of supplemental quercetin via MA of RCTs (5-days to 12-weeks duration) [91,92,93,94,95,96,97,98,99]. There were no dose–response MAs (Table S6).

When comparing the highest dietary intake with the lowest, kaempferol, but not myricetin or quercetin, reduced the risk of lung cancer by 23% (RR 0.77; 95% CI 0.62, 0.97) [84]. Supplemental quercetin improved a range of CVD risk factors, including systolic (MD −3.09 mmHg; 95% CI −4.83, −1.27) and diastolic blood pressure (MD −2.86 mmHg; 95% CI −5.09, −0.63) [93], CRP (MD −0.33 mg/L; 95% CI −0.50, −0.16) [94], VO2 max (MD 1.94%; 95% CI 0.30, 3.59) [97] and exercise performance (MD 2.82%; 95% CI 2.05, 3.58) [99]. RCT evidence for quercetin found no effect on blood lipids [91,93], glycemic or insulin metabolism [95], other measures of inflammation [96,98] or adiposity [92] (Table S6).

The strongest evidence for the health effect of flavonols and flavonols sub-classes was for improved blood pressure (cause-and-effect relationship established, large effect size, GRADE: low to high), cholesterol (cause-and-effect relationship established, small effect size, GRADE: low to high), blood glucose (cause-and-effect relationship established, small effect size, GRADE: medium) and risk of CVD or CHD mortality (dose–response relationship, very large effect size, GRADE: medium) and stroke (dose–response relationship, moderate to large effect size, GRADE: very low) (Figure 2).

### 2.9. Health Effects of White Bioactive Pigments in Fruits and Vegetables

The evidence for the health effects of white bioactive pigments were from MAs reporting on flavones. All *n* = 19 MAs for flavones were based on cohort data (1–24 years duration) as measured via the diet (*n* = 13 MAs) or mixed sources (*n* = 6 MAs), and two MAs were dose–response analyses (Tables S1 and S7). The highest category of flavones was associated with a decreased risk of all-cause (RR 0.86; 95% CI 0.80, 0.93) and CVD mortality (RR 0.85; 95% CI 0.75, 0.96) [84], breast cancer (RR 0.81; 95% CI 0.68, 0.96) [83,84], CHD (RR 0.94; 95% CI 0.89, 0.99) [74], esophageal cancer (OR 0.78; 95% CI 0.64, 0.95) [87], liver cancer (RR 0.49; 95% CI 0.30, 0.78) [84] and smoking-related cancer (OR 0.77; 95% CI 0.69, 0.85) [80] (Table S7). In dose–response MAs, for each 1 mg/day increase in flavones, the risk of CVD mortality decreased by 7% (RR 0.93; 95% CI 0.90, 0.97) [84]. No differences were found for hypertension [86], risk of CVD [76], risk of T2DM, or risk of colorectal [79], lung [82,84], ovarian [84], pancreatic [84] or prostate cancer [88] (Table S7).

The strongest evidence for the health effect of flavones was for decreased risk of all-cause and CVD mortality (dose–response relationship, moderate to large effect size, GRADE: very low to low), liver cancer (very large effect size, GRADE: medium) and smoking-related cancers (moderate effect size, GRADE: medium) (Figure 2).

### 2.10. Health Effects of Purple/Blue Bioactive Pigments in Fruits and Vegetables

Purple/blue bioactive pigments were contributed to by anthocyanidins, anthocyanins, proanthocyanidins and proanthocyanins.

#### 2.10.1. Anthocyanidins 

All *n* = 7 MAs examining anthocyanidins were based on cohort data derived from the diet (*n* = 2 MAs, 4–20 years duration) or mixed sources (*n* = 5 MAs, 4–16 years duration). The highest and lowest categories were not defined, but the single dose–response MA analyzed effects per 10 mg/day (Tables S1 and S8).

Higher anthocyanidin serum levels were associated with an 11% decrease in both all-cause (RR 0.89; 95% CI 0.85, 0.94) and CVD mortality (RR 0.89; 95% CI 0.83, 0.95). In dose–response MAs, for each 10 mg/day increase in anthocyanidins the risk of CVD mortality improved by 6% (RR 0.94; 95% CI 0.88, 0.99) [73]. Greater anthocyanidin intake was also associated with a 32% decreased risk of colorectal cancer (RR 0.68; 95% CI 0.56, 0.82) [79], 14% decreased risk of T2DM (HR 0.86; 95% CI 0.81, 0.91) [78], but a 12% increased risk of prostate cancer (RR 1.12; 95% CI 1.03, 1.21) [88] (Table S8). No association was found for smoking-related cancer [80].

#### 2.10.2. Anthocyanins

Most anthocyanin research was based on RCTs (*n* = 67 MAs) derived from diet (*n* = 19 MAs of 3-days to 6-weeks duration; dose not reported), mixed sources (*n* = 32 MAs of 4-h to 6-months duration, dose 1.3–1025 mg/day) or supplementation (*n* = 16 MAs of 1–96 weeks duration, dose 1.6–1323 mg/day). The *n* = 14 cohort MAs measured anthocyanins from the diet (*n* = 11 MAs, 1–24 years duration, dose not reported) or mixed sources (*n* = 3 MAs, 5–41 years, dose not reported) (Table S8).

The highest category of anthocyanin intake was associated with a decreased risk of CVD (RR 0.82; 95% CI 0.70, 0.96) [76], CHD (RR 0.90; 95% CI 0.83, 0.98) [74], CVD mortality (RR 0.92; 95% CI 0.87, 0.97) [100], hypertension (RR 0.92; 95% CI 0.88, 0.97) [86] and esophageal cancer (OR 0.60; 95% CI 0.49, 0.74) [87]. However, no association was found with risk of stroke [100], or multiple cancers including breast, liver, lung, pancreatic or gastric [83,84,101] (Table S8).

Thirty-three of the *n* = 67 (49%) RCT MAs reported improved inflammatory, oxidative, lipid, or glycemic markers (e.g., adiponectin, apolipoprotein A1/B, CRP, fasting glucose, HbA1c, HOMA-IR, LDL and HDL cholesterol, interleukin-6, TNF-alpha, triglycerides, see Table S8 for full list) [26,101,102,103,104], as well as vascular reactivity (SMD 0.77; 95% CI 0.37, 1.16) [105] and BMI (SMD −0.36 kg/m^2^; 95% CI −0.58, −0.13) [27]. No improvements were found for liver enzymes [106], uric acid, blood pressure [107], waist circumference [107], delayed onset muscle soreness [101] or vascular stiffness [105].

The strongest evidence for the health effect of anthocyanins and anthocyanidins was for improved inflammatory and oxidative stress biomarkers (cause-and-effect relationship established, small to large effect size, GRADE: very low to low), glycemic and insulinemic biomarkers (cause-and-effect relationship established, small effect size, GRADE: medium), lipid profiles and vascular function (cause-and-effect relationship established, small to large effect size, GRADE: very low to medium) and adiposity (cause-and-effect relationship established, small effect size, GRADE: low to medium) (Figure 2).

#### 2.10.3. Proanthocyanidins 

There were *n* = 11 MAs which reported on the effects of proanthocyanidins (*n* = 4 RCT MAs, *n* = 7 cohort MAs) (Table S1). Proanthocyanidin RCT MAs were all based on supplemental interventions of 100–400 mg/day delivered over 5 to 16 weeks. Cohort MAs were delivered over 4–16 years with unspecified categories of highest and lowest intakes, measured via diet (*n* = 3 MAs) or mixed sources (*n* = 4 MAs). One of the *n* = 7 cohort MAs was a dose–response analyses examining effects per 100 mg/day (Table S8).

The highest serum levels of proanthocyanidin compared with the lowest was associated with a 11% improvement in CVD mortality risk (RR 0.89; 95% CI 0.81, 0.97), but this was not significant in a dose–response analysis [84]. Higher status was also associated with a 28% decreased risk of colorectal cancer (RR 0.72; 95% CI 0.61, 0.85) [79]. No differences were found with risk of all cause-mortality, T2DM, breast cancer or esophageal cancer [73,78,87]. MAs of RCT evidence showed that supplemental proanthocyanidin (100–400 mg for 5–16 weeks) improved systolic (MD −4.60 mmHg; 95% CI −8.04, −1.16) and diastolic (MD −2.75 mmHg; 95% CI −5.09, −0.41) blood pressure and mean arterial pressure (MD −3.37 mmHg; 95% CI −6.72, −0.01), but not pulse pressure [108] (Table S8).

#### 2.10.4. Proanthocyanins

The two MAs of proanthocyanins were based on cohort data and measured the highest dietary intakes compared with the lowest for up to 16 years, and found an inverse association with risk of CVD (RR 0.83; 95% CI 0.73, 0.95) [76] and CHD (RR 0.78; 95%CI 0.65, 0.94) [74] (Table S8). 

The strongest evidence for the health effect of proanthocyanidins and proanthocyanins was for decreased blood and arterial pressure (large effect size, GRADE: high) (Figure 2).

### 2.11. Health Effects of Green Bioactive Pigments in Fruits and Vegetables

The health effects of consuming green bioactive pigments from FV were reported by single RCT and cohort evidence for chlorophyll. Ten of the seventeen health outcome measures reported for chlorophyll were based on RCT data (Sweden and Japan, 8–12 weeks of 0.7–3000 mg supplementation/day); the remaining seven were from cohort data (Netherlands, 9-years duration, highest undefined quintile) (Table S9). One RCT reported chlorophyll supplementation improved seasonal allergic rhinitis rescue medication scores (MD −3.09; 95% CI −5.96, −0.22) [109] and 3000 mg supplementation per day trended towards 1.5 kg weight loss; however, this appeared underpowered (*p* = 0.06, *n* = 36 participants) [110]. RCT evidence reported no effect on other measures of body composition or levels of insulin, glucose, or leptin [110]. Analysis of cohort data found no association between the highest intakes of chlorophyll and colorectal, colon or rectal cancer [111] (Table S9). 

### 2.12. Health Effects Unique to Each Bioactive Pigment

Many health outcomes were improved by three or more bioactive pigments, such as a decreased risk of all-cause mortality with the highest intakes of lycopene, beta-cryptoxanthin, beta-carotene, alpha-carotene, lutein and zeaxanthin, flavones and anthocyanin/anthocyanidin (Figure 2). Other improved health outcomes which were associated with three or more bioactive pigment colors were body weight; total cholesterol/lipid profiles; inflammatory biomarkers; CVD, CHD, CVD mortality; stroke; T2DM; and multiple cancers including breast, oral, lung, prostate, bladder, colorectal/colon/rectal and gastric (Figure 2; Tables S2–S9). 

Some health effects were unique to only one or two bioactive pigments or colors. Every FV bioactive pigment color had a single highly unique health effect which was not associated with any other pigment color, except red and yellow (Table 2). For example, only red bioactive pigments were associated with a decreased risk of pancreatic and laryngeal cancer, and only pale-yellow pigments were associated with improved exercise performance. All bioactive pigment colors also had other unique health effects that were associated with only two bioactive pigment colors. For example, decreased risk of cervical cancer was associated with only red and orange bioactive pigments, and decreased risk of esophageal cancer was only associated with white and blue/purple bioactive pigments (Table 2). 

Of the highly unique health effects (i.e., significant effect in a single color of FV), only four outcomes have been confirmed as being truly unique by being tested for association with three or more different bioactive pigments. Waist circumference, unique to carotenoids, was found not to be affected by anthocyanins nor flavonols; risk of hypertension, unique to anthocyanins, was found to have no association with flavones nor flavonols; risk of preeclampsia, unique to beta-carotene, was found to have no association with alpha-carotene, lutein nor lycopene; and risk of liver cancer, unique to flavones, was found to have no association with anthocyanins nor flavanols (Table 2 and Tables S2–S9). The remaining highly unique health effects reported in Table 2 were only tested for association with one or two bioactive pigments, and it is therefore unknown if they may be improved by other bioactive pigments also.

## 3. Discussion

This umbrella review synthesized an extensive body of evidence of the health effects of bioactive pigments from FV, representing 83 SLRS, 2847 original research studies (cohorts and RCTs), and data from over 37 million participants. This review found that many health outcomes were improved by consuming three or more bioactive pigments classes or subclasses, reinforcing the importance of total FV in the diet, irrespective of color [112,113]. However, this review found that color-associated variety in FV may confer additional health benefits beyond total FV intake. This finding is strengthened by the 2020 umbrella review by Wallace et al., which reported a non-linear relationship between higher total intake of FV and lower risk of chronic disease, with a threshold of about 5 serves or 800 g, beyond which further benefits were not observed [114]. Wallace et al., also reported additional benefits of certain types of vegetables which tended to have greater or more unique health effects, such as dark-green leafy vegetables and dark-colored berries. Whilst most dietary guidelines worldwide recommend consuming a variety of healthy food or a variety of FV specifically, only a limited number of national dietary guidelines specifically recommend FV should consumed in a variety of colors, including the Australian, Gabon, Polish, and several South and Central American countries (Argentina, Chile, Costa Rica, Dominican Republic, Grenada and Panama) [115]. This umbrella review provides novel evidence to support the revision of dietary guidelines internationally regarding optimal FV intake for population health.

Despite the magnitude of the data presented, the health benefits of bioactive pigments may extend beyond the current findings as treatment effects and outcomes not relevant to population health were excluded, and many unique health outcomes have not yet been tested via MA with any bioactive pigment, including dementia, depression and anxiety, and infectious disease. Some of the unique health effects were anticipated due to an understanding of the physiological actions, such as carotenoids which have a structural and functional role in vision [116]. There is emerging evidence that some health effects may be mediated through protein-flavonoid interactions [117,118], which may result in changes to enzyme activity, receptors, antibodies and transcription factors such as inhibition of xanthine oxidase [117,118]. As flavonoids include all colors of FV, further research on the flavonoid subclasses and minor subclasses is required to understand their mechanisms of action. Two-way interactions between polyphenols and microbiota may mediate some health effects through improvements in gastrointestinal barrier function, butyrate production and down regulation of genes associated with inflammation [119,120]. Although mechanisms of action for unique health effects beyond vision are less understood, the anti-inflammatory and antioxidant behavior of bioactive pigments are known to play a mechanistic role for many health outcomes [110,121,122]. Considering that all bioactive pigments demonstrate anti-inflammatory and antioxidant behavior, investigation into the mechanisms of action of the truly unique health effects of specific pigments is required.

Most outcomes presented had limited certainty that the pooled estimates represented the true effects (91% had a GRADE rating of very low or low), with the principal reason for downgrading confidence being observational study design. Although observational data provide a lower certainty in the evidence according to the GRADE system and do not imply causality, many dietary guidelines worldwide are underpinned by observational evidence, and the observational nature strengthens translation. Observational data are based on the usual intakes and behaviors of sample populations, thereby showing that the level of bioactive pigments required to have a significant health effect are achievable using existing food environments and systems. However, it must be acknowledged that many factors related to health, social and economic equity also determine the ability of an individual or population to consume the required bioactive pigment doses for a health effect [120,121]. This supposition is reinforced by the majority of included RCTs using supplemental bioactive pigments for intervention delivery, usually at doses unachievable through usual dietary intake, and are therefore unrealistic for translation to public health policy and health promotion activities. Observational data further strengthen the evidence by allowing the measurement of long-term outcomes such as disease incidence, which is often infeasible to measure in RCTs. 

There were some outcomes in this review where a combination of both observational and RCT evidence allowed for stronger conclusions to be drawn. Specifically, observational research demonstrates implementation feasibility and impact on disease outcomes, where RCT evidence demonstrates causation via the measurement of related biomarkers. For example, RCT evidence demonstrated that anthocyanins improved the CVD biomarkers of cholesterol, inflammation and blood glucose while cohort evidence confirmed a lower risk of CHD, hypertension, and CVD mortality. This alignment of observational and RCT data for anthocyanins is important; as other dietary strategies have a misalignment, for example, wholegrains are associated with decreased risk of CVD, yet RCT evidence is yet to confirm causality via association with related biomarkers [122]. Finally, the downgrading of certainty in the evidence due to the observational nature of the data may underestimate the strength of some findings as prospective cohort data are recognized to be the highest level of evidence for prognostic outcomes such as disease incidence [123].

While this review identified substantial evidence for the beneficial effects of bioactive pigments from FV on many health outcomes, both divergent and negative health effects were also identified. Divergent findings were expected, due to both variations in dose, measurement type (dietary versus supplemental versus serum), follow-up duration, study design (e.g., cohort versus case–control versus RCT), power and risk of bias, being reported within and across SLRs for a particular health effect. Additionally, other unreported sources of variation are also expected such as dataset quality, validity of the measurement tools, and sample characteristics. For example, *n* = 3 MAs based on RCT data from *n* = 3 different SLRs reported on the effect of anthocyanins on CRP; however, only one reported a significant effect. Differences between the three MAs possibly explaining the divergent findings include different measurement type, study duration, sample size, and dose. Of the *n* = 449 included MAs, *n* = 4 (<1%) reported negative health effects. Three of these negative health effects were based on the supplementation of beta-carotene and mortality [124,125]; one was based on dietary anthocyanin intake and risk of prostate cancer [88]. Due to the lack of a known and plausible mechanism, the negative effect of dietary anthocyanin intake on risk of prostate cancer is likely due to a type I error which is unable be addressed using the false discovery rate in an umbrella review study design. This explanation is supported by a small effect size and the *p*-value being higher than many other included significant findings (*p* = 0.011, where 60% of significant *p*-values were <0.01). In contrast, the negative effect of beta-carotene supplementation on all-cause and CVD-mortality may not be subject to error. Whilst effect sizes were small, *p*-values were highly significant, and 95% CIs were precise. Further, there is a plausible mechanism of action as well as precedence. Supplemental versus dietary antioxidants are suggested to have differing bioavailability, biomechanics and outcomes. For example, supplemental beta-carotene has been associated with pro-oxidation and increased risk of lung and stomach cancer [62,126], whereas dietary sources had no effect on cancer risk [43].

### 3.1. Implications for Future Research and Practice

This umbrella review provides a theoretical basis for improved health outcomes if color-associated FV variety of is increased by populations, and presents the first high-level evidence to substantiate existing health promotional messages which recommend community members to “eat a rainbow” of FV [15,127,128,129]. Translational and interventional research is required to improve translation to policy and practice. Valid and reliable diet quality assessment tools are required to facilitate the measurement and quantification of color associated FV variety and bioactive pigments from FV in both the clinical and research settings. Such tools will support the focus on color-associated FV variety and bioactive pigments as well as allow for interventional and observational research to directly measure association with health outcomes. To further support translation to practice, increased measurement of bioactive pigments in diverse FV is required so that FV rich in a particular bioactive pigment relevant to an individuals’ health goals can be recommended. Agricultural methods should continue be explored to maximize the bioactive pigment concentrations in various FV, and modifications to agricultural practices which have other goals (e.g., improved sustainability or yield) should also consider their impact on bioactive pigment concentrations. Additionally, the reductionist approach utilized in many food systems to decrease the variety of FV available for the purposes of streamlining production should be addressed via reintroduction of FV varieties no longer or rarely commercially available, e.g., yellow watermelon, white tomatoes, purple cauliflower or rainbow chard.

### 3.2. Limitations

The findings of this review have been strengthened by a strong study design and the utilization of validated and best-practice methodology. However, inherent limitations must be acknowledged to ensure conclusions are drawn in context. The findings of this review do not represent the entirety of the evidence for the effect of bioactive pigments on population-relevant health outcomes, as data were extracted for only the highest level of evidence available. Although the CCA methodology was used to prevent overlap, some overlap remains. For example, although original studies have low levels of overlap, it is possible that multiple original studies in the included SLRs drew upon the same datasets for their analyses. Additionally, while data from more than 37 million participants were extracted, a single participant may have contributed to two or more of the individual MAs (e.g., participant A included in MAs for the effect of both lutein and alpha-carotene on risk of T2DM). Conclusions are also limited to adults as no studies were found for children or adolescents. Each finding should be interpreted in the context of its’ GRADE rating as well as the SLR characteristics including study type, measurement type, risk of bias and length of follow-up.

## 4. Materials and Methods

The study protocol was prospectively registered with the International Prospective Register of Systematic Reviews (PROSPERO, https://www.crd.york.ac.uk/prospero/ (accessed on 4 October 2021)); registration number: CRD42021276401, and has been reported according to the Preferred Reporting Items for Systematic reviews and Meta-Analyses (PRISMA) 2020 Statement [130].

### 4.1. Characterization of Natural Pigments

This review focused on the health effects of natural pigments that are responsible for the visible colors of FV. Four major classes of natural plant pigments have demonstrated bioactivity in humans: carotenoids, flavonoids, betalains and chlorophylls. Within each major pigment class there are distinct subclasses that have been associated with typical colors in plants and some minor sub-classes that have received further examination in the literature (Table 1).

### 4.2. Eligibility Criteria

Studies were deemed eligible if they satisfied the PICOS (Participant, Intervention, Comparator, Outcome, Study design) eligibility criteria described in Table 3.

### 4.3. Search Strategy

The electronic databases PubMed, EMBASE, CINAHL and Cochrane Library (Reviews and CENTRAL) were searched from inception to 29 October 2021, without restrictions (Tables S10–S12). The systematic search strategy was designed to include a combination of both controlled vocabulary (e.g., MeSH terms) and title and abstract keywords. The keywords repeated the controlled vocabulary terms if relevant, plus additional keywords specific to the topic. The search strategy was designed in PubMed and then translated to the other databases using Polyglot Search Translator [131]. Reference lists from umbrella reviews were also examined to identify any further relevant studies. References were imported into Endnote X9 reference management software (version X9.3.3, Clarivate Analytics, Philadelphia, PA, USA) and deduplication performed. Remaining records were uploaded to Covidence, a web-based systematic review software for screening (https://www.covidence.org/ (accessed on 29 October 2021)).

### 4.4. Selection Process

Two researchers independently screened records for potential eligibility using the title and abstract (MB and SM/HM). Full texts were retrieved for all potentially eligible studies and two researchers (MB and SM) independently assessed each study against the full eligibility criteria (Table 3). Any discrepancies between researchers were resolved by consensus. The inter-rater reliability between reviewers at full text review is summarized in Table S13.

If multiple meta-analyses (MAs) examined the same pigment and health outcome, the degree of overlapping of studies included in eligible meta-analyzed groups were assessed by calculating the corrected covered area (CCA) for each type of intervention [132]. If a CCA was greater than 15% (very high overlapping), the meta-analysis (MA) with the largest number of total participants and/or the lowest statistical inconsistency/heterogeneity, as indicated by the I^2^ or Chi-squared statistic, was selected.

### 4.5. Data Extraction

The following data were extracted from each study: study and participant characteristics, bioactive pigment name and color, intervention (type, duration, and dose), comparator (type, duration, and dose), number of meta-analyzed studies/intervention groups, model, meta-analyzed outcome, original research study design, original studies risk of bias, sample size (intervention/case, comparator/control, and total), effect size, confidence interval, *p*-value, heterogeneity, publication bias and the Grading of Recommendations Assessment, Development and Evaluation (GRADE) quality rating (if reported). The GRADE approach considers the internal validity and external validity of all studies reporting on a particular outcome so as to judge confidence in the estimated effect across the body of research [133]. As few original authors applied GRADE, current investigators (SM and MB) completed GRADE assessments for each extracted MA using information provided in the relevant SLRs or collated from individual RCTs/cohort studies reported by the SLR. GRADE was not applied to outcomes reported by included RCTs/cohort studies due to insufficient number of studies. Data were extracted into a Microsoft Excel (Version 1908; Excel for Office 365) spreadsheet by one researcher (MB or SM), checked for accuracy by another researcher (MB or SM).

During data extraction, included studies were assessed for methodological quality using the Oxford University, Centre for Evidence-Based Medicine (CEBM) critical appraisal tool for systematic reviews [134], RCTs [135] or prognostic studies [136]. Internal validity was assessed by determining if the study met multiple criteria (yes, no, or unclear), with a ‘yes’ judgment indicating good study quality and reduced risk of bias. 

## 5. Conclusions

A potential benefit to population health was found to be associated with eating a rainbow of FV. High consumption of FV, irrespective of color or bioactive pigment concentration, was associated with many significant health improvements in adults; however, unique health benefits were found to be associated with individual bioactive pigments. Research to support both the measurement and recommendation of color-associated FV variety and specific bioactive pigments is needed to support translation to policy and practice.

## Figures and Tables

**Table 1:** Natural bioactive pigment classes and subclasses and the typical colors they produce in fruits and vegetables [16].

Pigment Class	Pigment Subclass	Pigment Minor Subclass	Typical Colors
Carotenoids	Lycopene	-	Red
	Beta-cryptoxanthin		
	Capsorubin		
	Capsanthin		
	Beta-carotene	-	Orange
	Alpha-carotene	-	Yellow
	Lutein		
	Zeaxanthin		
	Violaxanthin		
Flavonoids	Anthocyanins/	Cyanidin	Red, purple, blue
	anthocyanidins	Malvidin	
		Peonidin	
		Delphinidin	
		Pelargonidin	
		Petunidin	
	Aurones	Kaempferol	Pale yellow
	Chalcones	Quercetin	
	Flavonols	Myricetin	
	Flavones	Apigenin	White
		Luteolin	
		Isoetin	
	Tannins	Proanthocyanidins	Red, purple, blue, brown
		Proanthocyanins	
Betalains	Betacyanins	Betanin	Red, violet, orange, yellow
	Betaxanthin	Indicaxanthan	
		Vulgaxanthin	
Chlorophylls	Chlorophyll a and b	-	Green

**Table 2:** Unique health effects of bioactive pigment colors found in fruit or vegetables.

Bioactive Pigment Color	Highly Unique Health Effects ^a,c^	Unique Health Effects ^b,c^
Red/orange/yellow	↑ cognitive function (GRADE: medium) ↓ risk of IHD (GRADE: very low) ↑ HDL cholesterol (GRADE: high) ↓ waist circumference (GRADE: low to medium)	
Red		↓ risk of cervical cancer (GRADE: very low) ↓ risk of lung cancer (GRADE: very low) ↓ risk of pancreatic cancer (GRADE: very low) ↓ risk of pharyngeal cancer (GRADE: very low to medium) ↓ risk of hip fracture (GRADE: very low) ↓ risk of laryngeal cancer (GRADE: very low to medium)
Orange	↓ risk of preeclampsia (GRADE: very low) ↓ risk of total fracture (GRADE: very low) ↓ endometrial cancer (GRADE: very low)	↓ risk of non-Hodgkin lymphoma (GRADE: very low) ↓ risk of ovarian cancer (GRADE: very low) ↓ risk of cervical cancer (GRADE: very low) ↓ risk of pancreatic cancer (GRADE: very low) ↓ risk of cataract (GRADE: very low) ↓ risk of hip fracture (GRADE: very low) ↓ risk of laryngeal cancer (GRADE: very low to medium)
Yellow		↓ risk of non-Hodgkin lymphoma (GRADE: very low) ↓ risk of cataract (GRADE: very low) ↓ risk of pharyngeal cancer (GRADE: very low)
Pale-yellow	↑ exercise performance (GRADE: very low)	↓ risk of ovarian cancer (GRADE: low) ↓ risk of cervical cancer (GRADE: very low) ↓ blood pressure (GRADE: low to high) ↓ glycemic biomarkers (GRADE: medium) ↓ risk of smoking-related cancers (GRADE: very low)
White	↓ risk of liver cancer (GRADE: medium)	↓ risk of smoking-related cancers (GRADE: medium) ↓ risk of esophageal cancers (GRADE: very low)
Blue/purple	↓ risk of hypertension (GRADE: very low) ↓ oxidative stress biomarkers (GRADE: very low to low) ↓ insulinemic biomarkers (GRADE: medium) ↓ vascular function (GRADE: very low to medium) ↓ arterial pressure (GRADE: high)	↓ glycemic biomarkers (GRADE: medium) ↓ risk of esophageal cancers (GRADE: very low) ↓ blood pressure (GRADE: high)
Green	↓ seasonal rhinitis (GRADE: N/A)	

^a^ A health effect was considered highly unique if it was found to be associated with a single bioactive pigment color. ^b^ A health effect was considered unique if it was found to be associated with only two bioactive pigment colors. ^c^ GRADE working groups of evidence: high = further research is unlikely to change our confidence in the estimated effect; medium = further research is likely to have an important impact on our confidence in the estimate of effect and may change the estimate; low = further research is very likely to have an important impact on our confidence in the estimate of effect and is likely to change the estimate; very low = we are very uncertain about the estimate.

**Table 3:** PICOS Eligibility Criteria.

PICOS Elements	Inclusion Criteria	Exclusion Criteria
Participant/ population	Humans	Animal and in vitro
Intervention/ exposure	Natural pigments found in fruits and vegetables that contribute to their visible color (as described in Table 1). The pigment must be: (1) consumed through whole fruit or vegetable; (2) extract from fruits or vegetables; or (3) provided as a supplement derived from fruits or vegetables. SLRs which included a mix of natural and synthetic bioactive pigments, or the derivation of the bioactive pigment was not described, were included.	Pigments within pharmaceuticals or synthetic forms. Pigments sourced from non-fruit or vegetable foods (e.g., nuts, soy, tea). Nutrients or phytonutrients that are not pigments and do not contribute to the visible color of the FV, but may be high in concentration in FV of a particular color (e.g., folate in green fruits and vegetables). Pigments delivered as a co-intervention or administered via non-oral routes (e.g., topical, aromatherapy, moxibustion).
Comparator	Placebo, presence of the pigment versus no pigment, or varying levels of the pigment (comparison of high versus low).	No control or comparator group. Alternative intervention.
Outcome	Health-related outcomes relevant to population health including the prevention of disease and optimization of disease risk factors, general wellbeing, function (cognitive function, physical function, and exercise performance), growth and development in children, maternal and neonatal health.	Biomarkers of pigment intake, disease treatment (e.g., cancer treatment), in-born errors of metabolism, biomarkers not related to disease prevention.
Study design/ source	SLRs with MAs of RCTs and/or cohort studies. RCTs and/or cohort studies if no eligible SLRs available. Case–control studies were included if based on longitudinal data.	SLRs without MAs, cross-sectional studies, single arm interventions, narrative reviews, expert opinion articles, or consensus guidelines. Studies unable to be translated into English via Google Translate or manual translation by multilingual colleagues.

MA, meta-analysis; RCT, randomized controlled Trial; SLR, systematic literature review.

## Data Availability

The data presented in this study are openly available in the Dryad database.

## Supplementary Materials

The following supporting information can be downloaded at: https://www.mdpi.com/article/10.3390/molecules27134061/s1, Table S1. Summary of the number of SLRs and MAs included in this umbrella review, by bioactive pigment sourced from fruit or vegetables and heath outcome; Table S2. Meta-analysis results of included SLRs for the health effects of total carotenoid pigments found in fruit and vegetables; Table S3. Meta-analysis results of included SLRs for the health effects of red pigments found in fruit and vegetables; Table S4. Meta-analysis results of included SLRs for the health effects of orange pigments found in fruit and vegetables; Table S5. Meta-analysis results of included SLRs for the health effects of yellow pigments found in fruit and vegetables; Table S6. Meta-analysis results of included SLRs for the health effects of pale-yellow pigments found in fruit and vegetables; Table S7. Meta-analysis results of included SLRs for the health effects of white pigments found in fruit and vegetables; Table S8. Meta-analysis results of included SLRs for the health effects of purple/blue pigments found in fruit and vegetables; Table S9. Results of included RCTs and cohort studies for the health effects of green pigments found in fruit and vegetables; Table S10: PubMed systematic search strategy to identify umbrella reviews and systematic literature reviews of meta-analyses; Table S11. Systematic search strategy for EMBASE, CINAHL and Cochrane Library; Table S12. Secondary systematic search strategy for chlorophyll studies in PubMed, EMBASE, CINAHL and Cochrane Library; Table S13. Inter-rater reliability between reviewers at full text review.
