# Supplementary material for: Should We ‘Eat a Rainbow’? An Umbrella Review of the Health Effects of Colorful Bioactive Pigments in Fruits and Vegetables
Source: Molecules. 2022 Jun 24;27(13):4061. doi: 10.3390/molecules27134061 (PMC9268388; doi:10.3390/molecules27134061)
Supplement: Supplementary file 1 [file molecules-27-04061-s001.zip › molecules-1750065-supplementary.pdf]

Table S1. Summary of the number of SLRs and MAs included in this umbrella review, by bioactive pigment sourced from fruit or vegetables and heath outcome.

PIGMENT	NUMBER OF SLRS INCLUDED	NUMBER OF MA INCLUDED	NUMBER OF DOSE-RESPONSE MA INCLUDED	TOTAL NUMBER OF ORIGINAL RESEARCH STUDIES[a]	OUTCOME TYPES EVALUATED	NUMBER OF SIGNIFICANT HEALTH EFFECTS
**Major pigment classes**						
Carotenoids (Red/orange/ yellow)	4	12	0	62	Cognitive function (n=1 MA)<br>CVD (n=3 MA)<br>Obesity (n=8 MA)	Cognitive function (n=1 MA)<br>CVD (n=2 MA)<br>Obesity (n=5 MA)<br>**Total N=8 MA (67%)**
**Pigment sub-classes**						
Alpha-carotene (Yellow)	16	41	13	193	Bone health (n=1 MA)<br>Cancer (n=29 MA)<br>Eye health (n=2 MA)<br>Mortality (n=3 MA)<br>Nervous system (n=1 MA)<br>Pregnancy health (n=1 MA)<br>T2DM (n=4 MA)	Bone health (n=0 MA)<br>Cancer (n=14 MA)<br>Eye health (n=0 MA)<br>Mortality (n=3 MA)<br>Nervous system (n=0 MA)<br>Pregnancy health (n=0 MA)<br>T2DM (n=1 MA)<br>**Total N=18 MA (44%)**
Anthocyanidin (Red/purple/blue)	5	7	1	35	Cancer (n=3 MA)<br>CVD (n=1 MA)<br>Mortality (n=2 MA)<br>T2DM (n=1 MA)	Cancer (n=2[b] MA)<br>CVD (n=1 MA)<br>Mortality (n=2 MA)<br>T2DM (n=1 MA)<br>**Total N=6 MA (86%)**

Anthocyanin (Red/purple/blue)	18	81	0	729	Cancer (n=8 MA)<br>CVD (n=45 MA)<br>Exercise (n=26 MA)<br>Obesity (n=2 MA)	Cancer (n=1 MA)<br>CVD (n=26 MA)<br>Exercise (n=10 MA)<br>Obesity (n=2 MA)<br>**Total N=39 MA (48%)**
Beta-carotene (Orange)	33	74	16	478	Bone health (n=3 MA)<br>Cancer (n=44 MA)<br>Cognitive function (n=1 MA)<br>CVD (n=13 MA)<br>Eye health (n=2 MA)<br>Mortality (n=4 MA)<br>Nervous system (n=1 MA)<br>Pregnancy health (n=1 MA)<br>Respiratory health (n=1 MA)<br>T2DM (n=4 MA)	Bone health (n=2 MA)<br>Cancer (n=18 MA)<br>Cognitive function (n=0 MA)<br>CVD (n=5[c] MA)<br>Eye health (n=1 MA)<br>Mortality (n=4 MA)<br>Nervous system (n=0 MA)<br>Pregnancy health (n=1 MA)<br>Respiratory health (n=0 MA)<br>T2DM (n=3 MA)<br>**Total N=34 MA (46%)**
Beta-cryptoxanthin (Red)	15	33	7	127	Bone health (n=3 MA)<br>Cancer (n=18 MA)<br>CVD (n=2 MA)<br>Eye health (n=2 MA)<br>Mortality (n=3 MA)<br>Nervous system (n=1 MA)<br>T2DM (n=4 MA)	Bone health (n=1 MA)<br>Cancer (n=4 MA)<br>CVD (n=1 MA)<br>Eye health (n=0 MA)<br>Mortality (n=3 MA)<br>Nervous system (n=0 MA)<br>T2DM (n=1 MA)<br>**Total N=10 MA (30%)**
Flavones (White)	11	19	2	95	Cancer (n=11 MA)<br>CVD (n=4 MA)<br>Mortality (n=3 MA)<br>T2DM (n=1 MA)	Cancer (n=5 MA)<br>CVD (n=2 MA)<br>Mortality (n=2 MA)<br>T2DM (n=0 MA)<br>**Total N=9 MA (47%)**
Flavonols (Pale yellow)	17	33	4	279	Cancer (n=12 MA)	Cancer (n=5 MA)

					CVD (n=16 MA)<br>Mortality (n=3 MA)<br>Obesity (n=1 MA)<br>T2DM (n=1 MA)	CVD (n=13 MA)<br>Mortality (n=1 MA)<br>Obesity (n=0 MA)<br>T2DM (n=1 MA)<br>**Total N=20 MA (61%)**
Lutein (Yellow)	7	10	2	35	Cancer (n=3 MA)<br>CVD (n=2 MA)<br>Mortality (n=1 MA)<br>Nervous system (n=1 MA)<br>Pregnancy health (n=1 MA)<br>T2DM (n=2 MA)	Cancer (n=0 MA)<br>CVD (n=1 MA)<br>Mortality (n=0 MA)<br>Nervous system (n=0 MA)<br>Pregnancy health (n=0 MA)<br>T2DM (n=2 MA)<br>**Total N=3 MA (30%)**
Lutein and zeaxanthin (Yellow)	13	31	9	132	Bone health (n=1 MA)<br>Cancer (n=25 MA)<br>CVD (n=2 MA)<br>Eye health (n=2 MA)<br>Mortality (n=1 MA)	Bone health (n=0 MA)<br>Cancer (n=6 MA)<br>CVD (n=1 MA)<br>Eye health (n=1 MA)<br>Mortality (n=1 MA)<br>**Total N=9 MA (29%)**
Lycopene (Red)	25	65	11	382	Bone health (n=1 MA)<br>Cancer (n=33 MA)<br>CVD (n=20 MA)<br>Eye health (n=1 MA)<br>Mortality (n=4 MA)<br>Nervous system (n=1 MA)<br>Pregnancy health (n=1 MA)<br>T2DM (n=4 MA)	Bone health (n=0 MA)<br>Cancer (n=12 MA)<br>CVD (n=7 MA)<br>Eye health (n=0 MA)<br>Mortality (n=3 MA)<br>Nervous system (n=0 MA)<br>Pregnancy health (n=0 MA)<br>T2DM (n=2 MA)<br>**Total N=24 MA (37%)**
Zeaxanthin (Yellow)	2	3	1	6	T2DM (n=2 MA)<br>Mortality (n=1 MA)	T2DM (n=0 MA)<br>Mortality (n=0 MA)<br>**Total N=0 (0%)**

**Pigment minor sub-classes**						
Kaempferol (Pale yellow)	1	1	0	4	Cancer (n=1 MA)	Cancer (n=1 MA)<br>**Total N=1 MA (100%)**
Myricetin (Pale yellow)	1	1	0	2	Cancer (n=1 MA)	Cancer (n=0 MA)<br>**Total N=0 MA (0%)**
Quercitin (Pale yellow)	10	25	0	217	Cancer (n=1 MA)<br>CVD (n=20 MA)<br>Exercise (N=2 MA)<br>Obesity (N=2 MA)	Cancer (n=0 MA)<br>CVD (n=3 MA)<br>Exercise (N=2 MA)<br>Obesity (N=0 MA)<br>**Total N=5 (20%)**
Proanthocyanidin (Red/purple/blue)	5	11	1	62	Cancer (n=3 MA)<br>CVD (n=5 MA)<br>Mortality (n=2 MA)<br>T2DM (n=1 MA)	Cancer (n=1 MA)<br>CVD (n=4 MA)<br>Mortality (n=0 MA)<br>T2DM (n=0 MA)<br>**Total N=5 MA (46%)**
Proanthocyanin (Red/purple/blue)	2	2	0	9	CVD (n=2 MA)	CVD (n=2 MA)<br>**Total N=2 (100%)**

SLR, systematic literature review; MA, meta-analysis.

a. Indicates the number of intervention groups contributing to the cumulative meta-analyses. May include duplicate original research studies if an original research study was included in more than one meta-analysis.

b. One of the two significant cancer effects was a negative health effect.

c. Three of the five significant CVD effects were negative health effects.

Table S2. Meta-analysis results of included SLRs for the health effects of total carotenoid pigments found in fruit and vegetables.

AUTHOR	PIGMENT	DESIGN	TYPE	DOSE	DURATION	HEALTH OUTCOME	EFFECT SIZE	95% CI	P-VALUE
**Obesity & Type 2 diabetes**									
Yao et al.	Carotenoids	RCT	Supplement	1.2 – 60 mg	12 – 16 wk	BMI (kg/m^2)	-0.95[a]	-1.88, -0.01	<.001*
Yao et al.	Carotenoids	RCT	Supplement	1.2 – 60 mg	12 – 16 wk	Body weight (kg)	-2.34[a]	-3.80, -0.87	<.001*
Yao et al.	Carotenoids	RCT	Supplement	NR	12 – 16 wk	Fat ratio (%)	-0.75[a]	-1.76, 0.25	0.14
Yao et al.	Carotenoids	RCT	Supplement	NR	12 – 16 wk	HDL cholesterol (mg dL-1)	0.76[a]	0.10, 1.41	0.02*
Yao et al.	Carotenoids	RCT	Supplement	NR	12 – 16 wk	LDL cholesterol (mg dL-1)	-1.30[a]	-3.23, 0.63	0.19
Yao et al.	Carotenoids	RCT	Supplement	NR	12 – 16 wk	Total cholesterol (mg dL-1)	-2.10[a]	-3.20, -0.99	<.001*
Yao et al.	Carotenoids	RCT	Supplement	2 – 20 mg	12 – 16 wk	Waist circumference (cm)	-1.84[a]	-3.14, -0.54	<.001*
**Cognitive function**									
Davinelli et al.	Carotenoids	RCT	Mixed	0.5 – 50 mg	2 m – 18 y	Cognitive outcomes	0.14[a]	0.08, 0.20	<.001*
**Cardiovascular disease**									
Law & Morris	Carotenoids	Cohort	Serum	H vs L	12 – 14 y	Risk of IHD	0.57[b]	0.47, 0.69	<.001*
Law & Morris	Carotenoids	Cohort	Diet	H vs L	4 – 24 y	Risk of IHD	0.85[b]	0.77, 0.93	<.001*
Hajizadeh-Sharafabad et al.	Carotenoids	RCT	Mixed	6 – 30 mg	2 – 24 y	TNF-alpha (ph/mL)	-0.97[c]	-1.98, 0.03	0.06

BMI = body mass index; H vs L = highest dose (intervention) vs lowest dose (control); IHD = ischemic heart disease; NR = not reported; RCT = randomised controlled trial; TNF-alpha = tumor necrosis factor alpha; Total-C = total cholesterol; wk = weeks; y = years

[a] Standardized mean difference; [b] Risk ratio or relative risk; [c] Mean difference.

* Significant difference favoring the intervention.

;

Table S3. Meta-analysis results of included SLRs for the health effects of red pigments found in fruit and vegetables.

AUTHOR	PIGMENT	DESIGN	TYPE	DOSE	DURATION	HEALTH OUTCOME	EFFECT SIZE	95% CI	P-VALUE
					**Cancer**				
Wu et al.	Beta-cryptoxanthin	Cohort & CC	Diet	NR (Graphic only)	NR	Risk of bladder cancer	0.58[a]	0.36, 0.94	NR*
Wu et al.	Beta-cryptoxanthin	Cohort & CC	Diet	H vs L	NR	Risk of bladder cancer	0.90[a]	0.80, 1.02	NR
Wu et al.	Beta-cryptoxanthin	Cohort & CC	Serum	H vs L	NR	Risk of bladder cancer	0.64[a]	0.22, 1.86	NR
Hu et al.	Beta-cryptoxanthin	CC	Diet	200 µg/d vs 20 µg/d	1-5 y	Risk of breast cancer	0.95[b]	0.80, 1.13	0.54
Hu et al.	Beta-cryptoxanthin	Cohort	Diet	200 µg/d vs 20 µg/d	8-14 y	Risk of breast cancer	1.03[a]	0.96, 1.11	0.39
Panic et al.	Beta-cryptoxanthin	Cohort	Diet	H vs L	1-9 y	Risk of colon cancer	1.14[a]	0.62, 2.08	NR
Panic et al.	Beta-cryptoxanthin	Cohort	Diet	H vs L	1-9 y	Risk of colorectal cancer	1.99[a]	0.74, 1.34	NR
Panic et al.	Beta-cryptoxanthin	CC	Diet	H vs L	3-4 y	Risk of colorectal cancer	0.47[b]	0.12, 1.90	NR
Leoncini et al.	Beta-cryptoxanthin	CC	Diet	H vs L	1-8 y	Risk of larynx cancer	0.41[b]	0.33, 0.51	NR*
Gallicchio et al.	Beta-cryptoxanthin	Cohort & CC	Diet	>56-371 µg/1000kcal/d vs <5-<46 µg/d	6 -25 y	Risk of lung cancer	0.80[a]	0.72, 0.89	NR*
Gallicchio et al.	Beta-cryptoxanthin	Cohort & CC	Diet	DR: Per 10 µg/d	6-14 y	Risk of lung cancer	0.99[a]	0.98, 1.00	NR
Gallicchio et al.	Beta-cryptoxanthin	Cohort & CC	Serum	DR: Per 0.05 µmol/L	6-10 y	Risk of lung cancer	1.14[a]	0.75, 1.72	NR
Gallicchio et al.	Beta-cryptoxanthin	Cohort & CC	Serum	4->9 µg/dL vs <1.8-<4 µg/dL	6-12 y	Risk of lung cancer or lung cancer mortality	0.82[a]	0.40, 1.68	NR
Chen et al.	Beta-cryptoxanthin	Cohort & CC	Diet	H vs L	NR	Risk of non-Hodgkin lymphoma	0.87[a]	0.75, 1.01	NR
Chen et al.	Beta-cryptoxanthin	Cohort & CC	Diet	DR: Per 100 µg/d	NR	Risk of non-Hodgkin lymphoma	0.95[a]	0.89, 1.01	NR
Leoncini et al.	Beta-cryptoxanthin	CC	Diet	H vs L	1-12 y	Risk of oral cavity and pharynx cancer	0.46[b]	0.29, 0.74	NR*
Huang et al.	Beta-cryptoxanthin	Cohort & CC	Diet	H vs L	NR	Risk of pancreatic cancer	0.86[b]	0.67, 1.12	0.28
Panic et al.	Beta-cryptoxanthin	Cohort	Diet	H vs L	1-9 y	Risk of rectal cancer	0.68[b]	0.27, 1.74	NR
Wu et al.	Lycopene	Cohort & CC	Serum	H vs L	NR	Risk of bladder cancer	0.76[a]	0.39, 1.49	NR
Wu et al.	Lycopene	Cohort & CC	Diet	H vs L	NR	Risk of bladder cancer	0.99[a]	0.87, 1.12	NR
Hu et al.	Lycopene	Cohort	Diet	10,000 µg/d vs 2000 µg/d	2-14 y	Risk of breast cancer	0.99[a]	0.93, 1.06	0.77

Hu et al.	Lycopene	Cohort	Diet	DR: At 2000 µg/d	NR	Risk of breast cancer	1.00[a]	0.99, 1.01	0.88
Hu et al.	Lycopene	Cohort	Diet	DR: At 3000 µg/d	NR	Risk of breast cancer	1.00[a]	0.99, 1.02	0.88
Hu et al.	Lycopene	Cohort	Diet	DR: At 4000 µg/d	NR	Risk of breast cancer	1.00[a]	0.98, 1.02	0.88
Hu et al.	Lycopene	CC	Diet	3000 µg/d	NR	Risk of breast cancer	0.82[a]	0.74, 0.90	0.01*
Hu et al.	Lycopene	CC	Diet	2000 µg/d	NR	Risk of breast cancer	0.87[a]	0.82, 0.93	0.01*
Hu et al.	Lycopene	CC	Diet	10,000 µg/d vs 2000 µg/d	1-5 y	Risk of breast cancer	0.71[b]	0.56, 0.92	0.01*
Hu et al.	Lycopene	CC	Diet	4000 µg/d	NR	Risk of breast cancer	0.76[a]	0.68, 0.86	< 0.01*
Myung et al.	Lycopene	CC	Mixed	H vs L	1-16 y	Risk of cervical cancer	0.54[b]	0.39, 0.75	NR*
Panic et al.	Lycopene	Cohort	Diet	H vs L	1-9 y	Risk of colon cancer	0.94[a]	0.71, 1.24	NR
Panic et al.	Lycopene	CC	Diet	H vs L	1-3 y	Risk of colon cancer	0.95[b]	0.79, 1.15	NR
Panic et al.	Lycopene	Cohort	Diet	H vs L	1-9 y	Risk of colorectal cancer	1.08[a]	0.95, 1.23	NR
Panic et al.	Lycopene	CC	Diet	H vs L	1-6 y	Risk of colorectal cancer	0.92[b]	0.46, 1.83	NR
Zhou et al.	Lycopene	CC	Diet	H vs L	NR	Risk of gastric cancer	0.94[b]	0.73, 1.21	NR
Zhou et al.	Lycopene	Cohort	Diet	H vs L	NR	Risk of gastric cancer	0.80[b]	0.60, 1.07	NR
Leoncini et al.	Lycopene	CC	Diet	H vs L	1-8 y	Risk of larynx cancer	0.50[b]	0.28, 0.89	NR*
Gallicchio et al.	Lycopene	Cohort & CC	Diet	2035-18195 µg/d vs 132->4464 µg/d	6-25 y	Risk of lung cancer	0.86[a]	0.77, 0.97	NR*
Gallicchio et al.	Lycopene	Cohort & CC	Serum	>4.5 µg/dL vs <2 µg/dL	8-12 y	Risk of lung cancer	0.71[a]	0.51, 0.98	NR*
Gallicchio et al.	Lycopene	Cohort & CC	Diet	DR: Per 1000 µg/d	6-14 y	Risk of lung cancer	0.97[a]	0.94, 1.00	NR
Chen et al.	Lycopene	Cohort & CC	Diet	DR: Per 4000 µg/d	NR	Risk of non-Hodgkin lymphoma	0.96[a]	0.88, 1.06	NR
Chen et al.	Lycopene	Cohort & CC	Diet	H vs L	NR	Risk of non-Hodgkin lymphoma	0.99[a]	0.88, 1.12	NR
Leoncini et al.	Lycopene	CC	Diet	H vs L	1-12 y	Risk of oral cavity and pharynx cancer	0.74[b]	0.56, 0.98	NR*
Li and Xu a	Lycopene	Cohort & CC	Diet	H vs L	3 m-16 y	Risk of ovarian cancer	0.96[b]	0.86, 1.08	NR
Huang et al.	Lycopene	Cohort & CC	Diet	H vs L	NR	Risk of pancreatic cancer	0.84[b]	0.73, 0.97	0.02*
Rowles et al.	Lycopene	Cohort & CC	Diet	H vs L	3-13 y	Risk of advanced prostate cancer	0.93[a]	0.82, 1.07	0.59
Wang et al.	Lycopene	CC	Serum	H vs L	3-13 y	Risk of advanced prostate cancer	0.75[a]	0.44, 1.28	NR

Rowles et al.	Lycopene	Cohort & CC	Diet	H vs L	2-24 y	Risk of prostate cancer	0.88[a]	0.79, 0.99	0.02*
Rowles et al.	Lycopene	Cohort & CC	Serum	H vs L	2-22 y	Risk of prostate cancer	0.88[a]	0.79, 0.98	0.02*
Ilic et al.	Lycopene	RCT	Supplement	15-30 mg	4-6 m	Prostate specific antigen levels (ng/mL)	-0.34[c]	-2.01, 1.32	0.69
Panic et al.	Lycopene	Cohort	Diet	H vs L	1-9 y	Risk of rectal cancer	1.07[b]	0.54, 2.11	NR
Panic et al.	Lycopene	CC	Diet	H vs L	1-4 y	Risk of rectal cancer	0.82[b]	0.57, 1.16	NR
					**Cardiovascular disease**				
Hajizadeh-Sharafabad et al.	Beta-cryptoxanthin	RCT	Mixed	6 mg	12 wk	C-reactive protein (mg/L)	-0.35[c]	-0.54, -0.15	< 0.001*
Hajizadeh-Sharafabad et al.	Beta-cryptoxanthin	RCT	Mixed	6 mg	12 wk	IL-6 (pg/mL)	-0.55[c]	-1.13, 0.03	NR
Song et al.	Lycopene	Cohort & CC	Diet	H vs L	7-11 y	Risk of CHD	0.87[a]	0.76, 0.98	NR*
Song et al.	Lycopene	Cohort & CC	Serum	H vs L	11-13 y	Risk of CHD	0.79[a]	0.52, 1.07	NR
Cheng et al.	Lycopene	Cohort & CC	Mixed	H vs L	2-12 y	Risk of CVD	0.86[d]	0.77, 0.95	0.003*
Hajizadeh-Sharafabad et al.	Lycopene	RCT	Mixed	7-15 mg	4-16 wk	C-reactive protein (mg/L)	-1.23[c]	-3.53, 1.07	0.30
Tierney et al.	Lycopene	RCT	Mixed	6-50 mg	2-14 wk	Diastolic blood pressure (mmHg)	0.81[c]	-1.81, 3.44	0.54
Ried et al.	Lycopene	RCT	Mixed	4-45.6 mg	4 wk - 6 m	HDL cholesterol (mg/dL)	0.80[c]	-2.33, 3.93	0.62
Tierney et al.	Lycopene	RCT	Mixed	2-50 mg	1 d - 6 m	HDL cholesterol (mg/dL)	1.58[c]	-2.00, 5.16	0.39
Hajizadeh-Sharafabad et al.	Lycopene	RCT	Mixed	7-20 mg	4-16wk	IL-6 (pg/ml)	-1.08[c]	-2.03, -0.12	0.03*
Ried et al.	Lycopene	RCT	Mixed	4-45.6 mg	3 wk - 6 m	LDL cholesterol (mg/dL)	-4.63[c]	-9.24, -0.02	0.05
Tierney et al.	Lycopene	RCT	Mixed	2-50 mg	1 d - 6 m	LDL cholesterol (mg/dL)	-2.02[c]	-5.90, 1.86	0.31
Cheng et al.	Lycopene	Cohort & CC	Mixed	H vs L	4 - 13 y	Risk of stroke	0.74[d]	0.62, 0.89	0.001*
Li & Xu b	Lycopene	Cohort & CC	Mixed	H vs L	6-13 y	Risk of stroke	0.807[a]	0.68, 0.96	0.01*
Song et al.	Lycopene	Cohort & CC	Serum	H vs L	12-13 y	Risk of stroke	0.65[a]	0.42, 0.87	NR*
Song et al.	Lycopene	Cohort & CC	Diet	H vs L	6-11 y	Risk of stroke	0.83[a]	0.69, 0.96	NR*
Tierney et al.	Lycopene	RCT	Mixed	6-50 mg	2-14 wk	Systolic blood pressure (mmHg)	-0.64[c]	-3.23, 1.96	0.63

Hajizadeh-Sharafabad et al.	Lycopene	RCT	Mixed	7-20 mg	2-16 wk	TNF-alpha (pg/mL)	-1.83[c]	-4.10, 0.42	NR
Ried et al.	Lycopene	RCT	Mixed	4-45.6 mg	15 d - 6 m	Total cholesterol (mg/dL)	-0.87[c]	-4.12, 2.38	0.6
Tierney et al.	Lycopene	RCT	Mixed	2-50 mg	1 d - 6 m	Total cholesterol (mg/dL)	-0.32[c]	-5.70, 5.06	0.91
Ried et al.	Lycopene	RCT	Mixed	4-45.6 mg	4 wk - 6 m	Triglycerides (mg/dL)	3.21[c]	-10.5, 16.91	0.65
Tierney et al.	Lycopene	RCT	Mixed	2-50 mg	6 hours - 6 m	Triglycerides (mg/dL)	1.56[c]	-10.93, 14.05	0.81
**Mortality**									
Jayedi et al.	Beta-cryptoxanthin	Cohort	Mixed	H vs L	2-26 y	Risk of all-cause mortality	0.73[a]	0.58, 0.88	NR*
Jayedi et al.	Beta-cryptoxanthin	Cohort	Serum	H vs L	2-26 y	Risk of all-cause mortality	0.88[a]	0.79, 0.97	NR*
Jayedi et al.	Beta-cryptoxanthin	Cohort	Serum	DR: Per 0.1 μmol/L	2-26 y	Risk of all-cause mortality	0.94[a]	0.89, 0.99	NR*
Jayedi et al.	Lycopene	Cohort	Mixed	H vs L	2-26 y	Risk of all-cause mortality	0.72[a]	0.49, 0.95	NR*
Jayedi et al.	Lycopene	Cohort	Serum	H vs L	2-26 y	Risk of all-cause mortality	0.75[a]	0.54, 0.97	NR*
Jayedi et al.	Lycopene	Cohort	Serum	DR: Per 0.1 μmol/L	2-26 y	Risk of all-cause mortality	0.97[a]	0.94, 1.00	NR
Cheng et al.	Lycopene	Cohort & CC	Mixed	H vs L	8-18 y	Risk of mortality	0.63[d]	0.49, 0.81	< 0.001*
**Obesity & Type 2 diabetes**									
Jiang et al.	Beta-cryptoxanthin	Cohort	Serum	DR: Per 0.5 μmol/L	8-10 y	Risk of Type 2 diabetes	0.85[a]	0.76, 0.94	NR*
Jiang et al.	Beta-cryptoxanthin	Cohort	Serum	H vs L	8-10 y	Risk of Type 2 diabetes	0.80[a]	0.60, 1.06	NR
Jiang et al.	Beta-cryptoxanthin	Cohort	Diet	H vs L	10-23 y	Risk of Type 2 diabetes	0.82[a]	0.61, 1.10	NR
Jiang et al.	Beta-cryptoxanthin	Cohort	Diet	DR: Per 0.01 mg/d	10-23 y	Risk of Type 2 diabetes	0.99[a]	0.96, 1.03	NR
Jiang et al.	Lycopene	Cohort	Serum	DR: Per 0.5 μmol/L	8-10 y	Risk of Type 2 diabetes	0.83[a]	0.74, 0.92	NR*
Jiang et al.	Lycopene	Cohort	Serum	H vs L	8-10 y	Risk of Type 2 diabetes	0.85[a]	0.76, 0.96	NR*
Jiang et al.	Lycopene	Cohort	Diet	DR: Per 1 mg/d	7-23 y	Risk of Type 2 diabetes	1.00[a]	0.99, 1.01	NR
Jiang et al.	Lycopene	Cohort	Diet	H vs L	7-23 y	Risk of Type 2 diabetes	1.00[a]	0.90, 1.11	NR
**Bone health**									
Xu et al.	Beta-cryptoxanthin	Cohort	Mixed	H vs L	9-17 y	Risk of hip fracture	1.11[b]	0.97, 1.28	NR
Kim et al.	Beta-cryptoxanthin	Cohort & CC	Diet	H vs L	NR	Risk of hip fracture	0.72[b]	0.60, 0.87	<0.001*

Kim et al.	Beta-cryptoxanthin	Cohort & CC	Diet	H vs L	NR	Osteoporosis-related outcome	0.80[b]	0.65, 1.00	0.05
Xu et al.	Lycopene	Cohort	Mixed	H vs L	9-17 y	Risk of hip fracture	0.84[b]	0.69, 1.01	NR
					**Eye health**				
Jiang et al.	Beta-cryptoxanthin	Cohort	Diet	H vs L	5-15 y	Cataract incidence	0.95[a]	0.87, 1.05	0.33
Chong et al.	Beta-cryptoxanthin	Cohort	Mixed	H vs L	5-18 y	Incidence of early aged related macular degeneration	1.01[b]	0.85, 1.22	0.88
Chong et al.	Lycopene	Cohort	Mixed	H vs L	5-18 y	Incidence of early aged related macular degeneration	1.05[b]	0.88, 1.25	0.61
					**Neurodegenerative disease**				
Takeda et al.	Beta-cryptoxanthin	Cohort & CC	Diet	H vs L	NR	Risk of Parkinson's disease	0.96[b]	0.66, 1.40	0.84
Takeda et al.	Lycopene	Cohort & CC	Diet	H vs L	NR	Risk of Parkinson's disease	1.03[b]	0.64, 1.65	0.9
					**Pregnancy health**				
Cohen et al.	Lycopene	CC	Mixed	H vs L	9 m	Preeclampsia	-1.05[e]	-2.09, 0.00	0.05

CC = case control; CVD = cardiovascular disease; CHD = coronary heart disease; d = days; DR = dose response; H vs L = highest dose (intervention) vs lowest dose (control); IL-6 = interleuken-6; NR = not reported; RCT = randomised controlled trial; TNF-alpha = tumor necrosis factor alpha; Total-C = total cholesterol; wk = weeks; y = years.

[a] Risk Ratio or relative risk; [b] Odds Ratio; [c] Mean difference; [d] Hazard Ratio; [e] Standardized mean difference.

* Significant difference favoring the intervention.

Table S4. Meta-analysis results of included SLRs for the health effects of orange pigments found in fruit and vegetables.

AUTHOR	PIGMENT	DESIGN	TYPE	DOSE	DURATION	HEALTH OUTCOME	EFFECT SIZE	95% CI	P-VALUE
					**Cancer**				
Wu et al.	Beta-carotene	Cohort & CC	Serum	DR: Graphic only	NR	Risk of bladder cancer	0.73[b]	0.57, 0.94	NR*
Wu et al.	Beta-carotene	Cohort & CC	Serum	H vs L	NR	Risk of bladder cancer	0.67[b]	0.28, 1.62	NR
Wu et al.	Beta-carotene	Cohort & CC	Supplement	600-1991 µg/d	NR	Risk of bladder cancer	0.94[b]	0.70, 1.26	NR
Wu et al.	Beta-carotene	Cohort & CC	Diet	H vs L	NR	Risk of bladder cancer	0.95[b]	0.88, 1.03	NR
Hu et al.	Beta-carotene	Cohort	Diet	DR: 5000 µg/d	NR	Risk of breast cancer	0.93[b]	0.89, 0.97	<0.01*
Hu et al.	Beta-carotene	Cohort	Diet	DR: 3000 µg/d	NR	Risk of breast cancer	0.96[b]	0.93, 0.98	<0.01*
Hu et al.	Beta-carotene	Cohort	Diet	DR: 2000 µg/d	NR	Risk of breast cancer	0.97[b]	0.96, 0.99	<0.01*
Hu et al.	Beta-carotene	Cohort	Mixed	7000 µg/d vs 1500 µg/d	4-14 y	Risk of breast cancer	0.95[b]	0.90, 1.01	0.08
Hu et al.	Beta-carotene	CC	Mixed	DR: 5000 µg/d	NR	Risk of breast cancer	0.76[b]	0.70, 0.82	0.01*
Hu et al.	Beta-carotene	CC	Mixed	DR: 3000 µg/d	NR	Risk of breast cancer	0.85[b]	0.81, 0.88	0.01*
Hu et al.	Beta-carotene	CC	Mixed	DR: 2000 µg/d	NR	Risk of breast cancer	0.89[b]	0.87, 0.92	0.01*
Hu et al.	Beta-carotene	CC	Diet	7000 µg/d vs 1500 µg/d	1-5 y	Risk of breast cancer	0.75[a]	0.67, 0.85	<0.01*
Druesne-Pecollo et al.	Beta-carotene	RCT	Supplement	20-25 mg/d	2-13 y	Risk of cancer (any type)	1.02[b]	0.97, 1.07	0.45
Myung et al.	Beta-carotene	CC	Mixed	H vs L	1-16 y	Risk of cervical cancer	0.68[a]	0.55, 0.84	NR*
Panic et al.	Beta-carotene	Cohort	Diet	H vs L	1-9 y	Risk of colon cancer	0.88[b]	0.72, 1.07	NR
Panic et al.	Beta-carotene	CC	Diet	H vs L	3-8 y	Risk of colon cancer	0.78[a]	0.50, 1.24	NR
Druesne-Pecollo et al.	Beta-carotene	RCT	Supplement	20-25 mg/d	2-13 y	Risk of colorectal cancer	0.99[b]	0.83, 1.18	0.89
Panic et al.	Beta-carotene	Cohort	Diet	H vs L	1-9 y	Risk of colorectal cancer	0.98[b]	0.87, 1.11	NR
Panic et al.	Beta-carotene	CC	Diet	H vs L	2-8 y	Risk of colorectal cancer	0.64[a]	0.38, 1.08	NR
Xu et al.	Beta-carotene	CC	Diet	H vs L	4 y	Risk of colorectal cancer	0.47[b]	0.24, 0.91	NR*
Bandera et al.	Beta-carotene	CC	Diet	DR: Per 1000 µg/1000kcal	NR	Risk of endometrial cancer	0.88[a]	0.79, 0.98	<0.001*
Druesne-Pecollo et al.	Beta-carotene	RCT	Supplement	20-25 mg/d	2-13 y	Risk of gastric cancer	1.16[b]	0.78, 1.73	0.47
Zhou et al.	Beta-carotene	Cohort	Diet	H vs L	NR	Risk of gastric cancer	0.74[a]	0.61, 0.91	NR*

Zhou et al.	Beta-carotene	CC	Diet	H vs L	NR	Risk of gastric cancer	0.52[a]	0.46, 0.59	NR*
Leoncini et al.	Beta-carotene	CC	Diet	H vs L	3-8 y	Risk of larynx cancer	0.43[a]	0.24, 0.77	NR*
Druesne-Pecollo et al.	Beta-carotene	RCT	Supplement	20-25 mg/d	2-13 y	Risk of lung cancer	1.09[b]	0.94, 1.26	0.24
Gallicchio et al.	Beta-carotene	Cohort & CC	Serum	DR: Per 0.1 µmol/L	6-14 y	Risk of lung cancer	0.95[b]	0.87, 1.03	NR
Gallicchio et al.	Beta-carotene	Cohort & CC	Diet	DR: Per 500 µg/d	6-24 y	Risk of lung cancer	0.99[b]	0.98, 1.00	NR
Gallicchio et al.	Beta-carotene	RCT	Supplement	20-50 mg/d	2-13 y	Risk of lung cancer or lung cancer mortality	1.10[b]	0.89, 1.36	NR
Gallicchio et al.	Beta-carotene	Cohort & CC	Serum	>16->120 µg/dL vs <7-<30 µg/dL	6-14 y	Risk of lung cancer or lung cancer mortality	0.84[b]	0.66, 1.07	NR
Gallicchio et al.	Beta-carotene	Cohort & CC	Diet	2473 to 8950 µg/d vs <977 to <3152 µg/d	6-25 y	Risk of lung cancer or lung cancer mortality	0.92[b]	0.83, 1.01	NR
Zhang et al.	Beta-carotene	Cohort & CC	Mixed	H vs L	NR	Risk of melanoma	0.87[a]	0.62, 1.20	NR
Chen et al.	Beta-carotene	Cohort & CC	Diet	H vs L	NR	Risk of non-Hodgkin lymphoma	0.80[b]	0.68, 0.94	NR*
Leoncini et al.	Beta-carotene	CC	Diet	H vs L	5-8 y	Risk of oral cavity and pharynx cancer	0.67[a]	0.29, 1.53	NR
Leoncini et al.	Beta-carotene	CC	Diet	H vs L	1-3 y	Risk of oral cavity cancer	0.54[a]	0.37, 0.80	NR*
Huncharket et al.	Beta-carotene	Cohort & CC	Diet	H vs L	NR	Risk of ovarian cancer	0.84[b]	0.75, 0.94	NR*
Druesne-Pecollo et al.	Beta-carotene	RCT	Supplement	20-25 mg/d	2-13 y	Risk of pancreatic cancer	0.94[b]	0.41, 2.14	0.89
Huang et al.	Beta-carotene	Cohort & CC	Diet	H vs L	NR	Risk of pancreatic cancer	0.78[a]	0.66, 0.92	0.003*
Druesne-Pecollo et al.	Beta-carotene	RCT	Supplement	20-25 mg/d	2-13 y	Risk of prostate cancer	1.02[b]	0.93, 1.12	0.71
Wang et al.	Beta-carotene	CC	Serum	H vs L	3-20 y	Risk of prostate cancer	0.96[b]	0.81, 1.14	0.65
Wang et al.	Beta-carotene	Cohort & CC	Diet	H vs L	6-20 y	Risk of prostate cancer	0.90[b]	0.81, 1.01	0.07
Jiang et al.	Beta-carotene	RCT	Mixed	20-50 mg	4-12 y	Incidence of prostate cancer	0.97[b]	0.90, 1.05	0.51
Panic et al.	Beta-carotene	CC	Diet	H vs L	4-8 y	Risk of rectal cancer	1.13[a]	0.85, 1.51	NR
Panic et al.	Beta-carotene	Cohort	Diet	H vs L	1-9 y	Risk of rectal cancer	1.05[a]	0.80, 1.37	NR
				**Cardiovascular disease**					
Mente et al.	Beta-carotene	RCT	Supplement	NR	NR	Risk of CHD	1.01[b]	0.92, 1.09	NR

Mente et al.	Beta-carotene	Cohort	Diet	H vs L	NR	Risk of CHD	0.73[b]	0.65, 0.82	NR*
Ye et al.	Beta-carotene	Cohort	Mixed	H vs L	4-24 y	Risk of CHD	0.78[b]	0.53, 1.04	NR
Ye et al.	Beta-carotene	Cohort	Mixed	DR: Per 1 mg/d	4-24 y	Risk of CHD	1.00[b]	0.88, 1.14	NR
Seyedrezazadeh et al.	Beta-carotene	CC	Diet	NR	NR	Risk of COPD	0.99[b]	0.99, 1.00	0.9
Fortmann et al.	Beta-carotene	RCT	Supplement	50 mg	4-12 y	Risk of CVD	1.01[b]	0.93, 1.09	NR
Hajizadeh-Sharafabad et al.	Beta-carotene	RCT	Mixed	10-15 mg	8 wk	C-reactive protein (units NR)	-2.31[c]	-5.67, 1.04	NR
Vivekananthan et al.	Beta-carotene	RCT	Supplement	80-200 mg	2-12 y	Risk of all-cause cerebrovascular accident	1.00[a]	0.91, 1.09	0.92
**Mortality**									
Fortmann et al.	Beta-carotene	RCT	Supplement	50 mg	4-16 y	Risk of all-cause mortality	1.05[b]	1.02, 1.08	NR**
Vivekananthan et al.	Beta-carotene	RCT	Supplement	60-200 mg	2-12 y	Risk of all-cause mortality	1.07[a]	1.02, 1.11	0.003**
Jayedi et al.	Beta-carotene	Cohort	Serum	H vs L	2-26 y	Risk of all-cause mortality	0.63[b]	0.57, 0.70	NR*
Jayedi et al.	Beta-carotene	Cohort	Mixed	H vs L	2-26 y	Risk of all-cause mortality	0.82[b]	0.77, 0.86	NR*
Jayedi et al.	Beta-carotene	Cohort	Mixed	DR: Per 1 mg/d	2-26 y	Risk of all-cause mortality	0.95[b]	0.92, 0.99	NR*
Jayedi et al.	Beta-carotene	Cohort	Serum	DR: Per 0.1 µmol/L	2-26 y	Risk of all-cause mortality	0.96[b]	0.94, 0.98	NR*
Jayedi et al.	Beta-carotene	Cohort & CC	Serum	H vs L	4-26 y	Risk of CVD mortality	0.68[b]	0.52, 0.83	NR*
Jayedi et al.	Beta-carotene	Cohort & CC	Mixed	H vs L	4-14 y	Risk of CVD mortality	0.89[b]	0.73, 1.05	NR
Jayedi et al.	Beta-carotene	Cohort & CC	Mixed	DR: Per 0.1 µmol/L	4-14 y	Risk of CVD mortality	0.93[b]	0.84, 1.01	NR
Vivekananthan et al.	Beta-carotene	RCT	Supplement	60-200 mg	4-12 y	Risk of CVD mortality	1.10[a]	1.03, 1.17	0.003**
**Obesity & Type 2 diabetes**									
Jiang et al.	Beta-carotene	Cohort	Serum	H vs L	7-20 y	Risk of Type 2 diabetes	0.60[b]	0.46, 0.78	NR*
Jiang et al.	Beta-carotene	Cohort	Diet	H vs L	7-23 y	Risk of Type 2 diabetes	0.78[b]	0.70, 0.87	NR*
Jiang et al.	Beta-carotene	Cohort	Serum	DR: Per 0.5 µmol/L	7-10 y	Risk of Type 2 diabetes	0.65[b]	0.48, 0.89	NR*
Jiang et al.	Beta-carotene	Cohort	Diet	DR: Per 1 mg/d	7-23 y	Risk of Type 2 diabetes	0.95[b]	0.90, 1.01	NR
**Bone health**									
Charkos et al.	Beta-carotene	Cohort & CC	Diet	H vs L	NR	Risk of total fracture	0.63[b]	0.52, 0.77	<0.001*

Xu et al.	Beta-carotene	Cohort & CC	Mixed	H vs L	NR	Risk of hip fracture	0.72[a]	0.54, 0.95	NR*
Zhang et al.	Beta-carotene	Cohort & CC	Mixed	H vs L	NR	Risk of total fracture	1.07[b]	0.97, 1.17	NR
**Eye health**									
Chong et al.	Beta-carotene	Cohort	Mixed	H vs L	5-18 y	Incidence of early aged related macular degeneration	1.04[a]	0.86, 1.25	0.71
Jiang et al.	Beta-carotene	Cohort	Diet	H vs L	5-15 y	Cataract incidence	0.90[b]	0.83, 0.99	0.023*
**Neurodegenerative disease**									
Li et al.	Beta-carotene	CC	Diet	H vs L	NR	Risk of Alzheimer's disease	0.88[b]	0.73, 1.03	NR
Takeda et al.	Beta-carotene	Cohort & CC	Diet	H vs L	NR	Risk of Parkinson’s disease	0.91[a]	0.70, 1.20	0.52
**Pregnancy health**									
Cohen et al.	Beta-carotene	Cohort & CC	Mixed	H vs L	9 m	Preeclampsia	-0.40[d]	-0.72, -0.08	0.01*

CC = case control; CVD = cardiovascular disease; CHD = coronary heart disease; COPD = chronic obstructive pulmonary disease; DR = dose response; H vs L = highest dose (intervention) vs lowest dose (control); IHD = ischemic heart disease; m = months; NR = not reported; RCT = randomised controlled trial; wk = weeks; y = years

[a] Odds ratio; [b] Risk ratio or relative risk; [c] Mean difference; [d] Standardized mean difference.

* Significant difference favoring the intervention

** Significant difference favoring control

Table S5. Meta-analysis results of included SLRs for the health effects of yellow pigments found in fruit and vegetables.

AUTHOR	PIGMENT	DESIGN	TYPE	DOSE	DURATION	HEALTH OUTCOME	EFFECT SIZE	95% CI	P-VALUE
				**Cancer**					
Wu et al.	Alpha-carotene	Cohort & CC	Serum	H vs L	NR	Risk of bladder cancer	0.52[a]	0.21, 1.27	NR
Wu et al.	Alpha-carotene	Cohort & CC	Diet	H vs L	NR	Risk of bladder cancer	0.94[a]	0.80, 1.09	NR
Wu et al.	Alpha-carotene	Cohort & CC	Serum	DR: Graphic only	NR	Risk of bladder cancer	0.24[a]	0.08, 0.67	NR*
Hu et al.	Alpha-carotene	Cohort	Diet	DR: 1500 µg/d	NR	Risk of breast cancer	0.91[a]	0.87, 0.96	0.01*
Hu et al.	Alpha-carotene	Cohort	Diet	DR: 1000 µg/d	NR	Risk of breast cancer	0.94[a]	0.91, 0.98	0.01*
Hu et al.	Alpha-carotene	Cohort	Diet	DR: 500 µg/d	NR	Risk of breast cancer	0.97[a]	0.95, 0.99	0.01*
Hu et al.	Alpha-carotene	Cohort	Diet	2000 µg/d	8-14 y	Risk of breast cancer	0.91[a]	0.85, 0.98	0.01*
Hu et al.	Alpha-carotene	CC	Diet	DR: 1500 µg/d	NR	Risk of breast cancer	0.75[a]	0.62, 0.90	0.01*
Hu et al.	Alpha-carotene	CC	Diet	DR: 1000 µg/d	NR	Risk of breast cancer	0.82[a]	0.73, 0.93	0.01*
Hu et al.	Alpha-carotene	CC	Diet	DR: 500 µg/d	NR	Risk of breast cancer	0.91[a]	0.85, 0.97	0.01*
Hu et al.	Alpha-carotene	CC	Diet	2000 µg/d	1-2 y	Risk of breast cancer	0.82[b]	0.70, 0.97	0.02*
Leoncini et al.	Alpha-carotene	CC	Diet	H vs L	1-8 y	Risk of cancer of the larynx	0.46[b]	0.20, 1.06	NR
Panic et al.	Alpha-carotene	Cohort	Diet	H vs L	1-9 y	Risk of colon cancer	1.00[a]	0.84, 1.18	NR
Panic et al.	Alpha-carotene	Cohort	Diet	H vs L	1-9 y	Risk of colorectal cancer	1.05[a]	0.92, 1.21	NR
Panic et al.	Alpha-carotene	CC	Diet	H vs L	3-5 y	Risk of colorectal cancer	0.58[b]	0.33, 1.03	NR
Zhou et al.	Alpha-carotene	Cohort	Diet	H vs L	NR	Risk of gastric cancer	0.79[b]	0.59, 1.07	NR
Zhou et al.	Alpha-carotene	CC	Diet	H vs L	NR	Risk of gastric cancer	0.58[b]	0.44, 0.76	NR*
Chen et al.	Alpha-carotene	Cohort & CC	Diet	H vs L	NR	Risk of non-Hodgkin lymphoma	0.87[a]	0.78, 0.97	NR*
Chen et al.	Alpha-carotene	Cohort & CC	Diet	DR: Per 1000 µg/d	NR	Risk of non-Hodgkin lymphoma	0.87[a]	0.78, 0.97	NR*
Gallicchio et al.	Alpha-carotene	Cohort & CC	Serum	DR: Per 0.05 µmol/L	6-10 y	Risk of lung cancer	0.84[a]	0.44, 0.59	NR
Gallicchio et al.	Alpha-carotene	Cohort & CC	Diet	>881 to 1810 µg/d vs <180 to <558 µg/d	6-25 y	Risk of lung cancer	0.89[a]	0.79, 1.00	NR
Gallicchio et al.	Alpha-carotene	Cohort & CC	Diet	DR: Per 200 µg/d	6-14 y	Risk of lung cancer	0.99[a]	0.97, 1.01	NR

Gallicchio et al.	Alpha-carotene	Cohort & CC	Serum	>1 to >5 µg/dL vs <1 to <2 µg/dL	6-12 y	Risk of lung cancer or lung cancer mortality	0.89[a]	0.59, 1.33	NR
Leoncini et al.	Alpha-carotene	CC	Diet	H vs L	1-12 y	Risk of oral cavity and pharynx cancer	0.57[b]	0.41, 0.79	NR*
Wang et al.	Alpha-carotene	CC	Serum	H vs L	8-13 y	Risk of advanced prostate cancer	1.07[a]	0.75, 1.52	NR
Huang et al.	Alpha-carotene	Cohort & CC	Diet	H vs L	NR	Risk of pancreatic cancer	0.88[b]	0.66, 1.18	0.41
Wang et al.	Alpha-carotene	Cohort & CC	Diet	H vs L	6-20 y	Risk of prostate cancer	0.87[a]	0.76, 0.99	0.04*
Wang et al.	Alpha-carotene	CC	Serum	H vs L	4-20 y	Risk of prostate cancer	0.91[a]	0.72, 1.15	0.44
Panic et al.	Alpha-carotene	Cohort	Diet	H vs L	1-9 y	Risk of rectal cancer	1.02[b]	0.77, 1.35	NR
Zhou et al.	Lutein	CC	Diet	H vs L	NR	Risk of gastric cancer	0.89[b]	0.68, 1.15	NR
Gallicchio et al.	Lutein	Cohort	Diet	4041-3701 µg/d vs 1413-1736 µg/d	10-12 y	Risk of lung cancer	0.90[a]	0.74, 1.10	NR
Gallicchio et al.	Lutein	Cohort & CC	Diet	DR: Per 500 µg/d	10-12 y	Risk of lung cancer	0.98[a]	0.97, 1.10	NR
Wu et al.	Lutein & Zeaxanthin	Cohort & CC	Serum	H vs L	NR	Risk of bladder cancer	0.53[a]	0.33, 0.84	NR*
Wu et al.	Lutein & Zeaxanthin	Cohort & CC	Diet	H vs L	NR	Risk of bladder cancer	0.91[a]	0.76, 1.10	NR
Wu et al.	Lutein & Zeaxanthin	Cohort & CC	Serum	DR: Graphic only	NR	Risk of bladder cancer	0.44[a]	0.28, 0.67	NR*
Hu et al.	Lutein & Zeaxanthin	Cohort	Diet	5000 µg/d vs 1000 µg/d	2-14 y	Risk of breast cancer	0.94[a]	0.87, 1.02	0.13
Hu et al.	Lutein & Zeaxanthin	Cohort	Diet	2000 µg/d vs 300 µg/d	NR	Risk of breast cancer	1.00[a]	0.94, 1.08	0.91
Hu et al.	Lutein & Zeaxanthin	Cohort	Diet	DR: 3000 µg/d	NR	Risk of breast cancer	1.00[a]	0.91, 1.12	0.91
Hu et al.	Lutein & Zeaxanthin	Cohort	Diet	DR: 4000 µg/d	NR	Risk of breast cancer	1.01[a]	0.82, 1.24	0.96
Hu et al.	Lutein & Zeaxanthin	CC	Diet	2000 µg/d vs 300 µg/d	NR	Risk of breast cancer	0.88[a]	0.84, 0.92	0.01*
Hu et al.	Lutein & Zeaxanthin	CC	Diet	DR: 3000 µg/d	NR	Risk of breast cancer	0.83[a]	0.77, 0.89	<0.01*
Hu et al.	Lutein & Zeaxanthin	CC	Diet	5000 µg/d vs 1000 µg/d	1-5 y	Risk of breast cancer	0.79[b]	0.66, 0.94	0.01*
Hu et al.	Lutein & Zeaxanthin	CC	Diet	DR: 4000 µg/d	NR	Risk of breast cancer	0.88[a]	0.75, 1.05	0.15
Panic et al.	Lutein & Zeaxanthin	Cohort	Diet	H vs L	1-9 y	Risk of colon cancer	0.92[a]	0.77, 1.09	NR
Panic et al.	Lutein & Zeaxanthin	CC	Diet	H vs L	3-4 y	Risk of colon cancer	0.89[b]	0.77, 1.03	NR
Panic et al.	Lutein & Zeaxanthin	Cohort	Diet	H vs L	1-9 y	Risk of colorectal cancer	1.05[a]	0.91, 1.20	NR
Panic et al.	Lutein & Zeaxanthin	CC	Diet	H vs L	3-6 y	Risk of colorectal cancer	0.78[b]	0.56, 1.09	NR

Zhou et al.	Lutein & Zeaxanthin	Cohort	Diet	H vs L	NR	Risk of gastric cancer	0.95[b]	0.77, 1.18	NR
Leoncini et al.	Lutein & Zeaxanthin	CC	Diet	H vs L	1-8 y	Risk of larynx cancer	0.60[b]	0.27, 1.32	NR
Gallicchio et al.	Lutein & Zeaxanthin	Cohort	Diet	>1815 to 3857 µg/d vs <775 to 1388 µg/d	6-25 y	Risk of lung cancer	0.89[a]	0.79, 1.10	NR
Gallicchio et al.	Lutein & Zeaxanthin	Cohort & CC	Diet	DR: Per 500 µg/d	6-14 y	Risk of lung cancer	0.97[a]	0.84, 1.12	NR
Gallicchio et al.	Lutein & Zeaxanthin	Cohort & CC	Serum	DR: Per 0.1 µmol/L	6-10 y	Risk of lung cancer	0.98[a]	0.90, 1.06	NR
Gallicchio et al.	Lutein & Zeaxanthin	Cohort & CC	Serum	>40 to >60 µg/dL vs <24 to <44 µg/dL	6-12 y	Risk of lung cancer or lung cancer mortality	0.95[a]	0.67, 1.36	NR
Chen et al.	Lutein & Zeaxanthin	Cohort & CC	Diet	H vs L	NR	Risk of non-Hodgkin lymphoma	0.82[a]	0.69, 0.97	NR*
Leoncini et al.	Lutein & Zeaxanthin	CC	Diet	H vs L	1-12 y	Risk of oral cavity and pharynx cancer	0.51[b]	0.22, 1.18	NR
Huang et al.	Lutein & Zeaxanthin	Cohort & CC	Diet	H vs L	NR	Risk of pancreatic cancer	0.80[b]	0.61, 1.05	0.10
Panic et al.	Lutein & Zeaxanthin	Cohort	Diet	H vs L	1-9 y	Risk of rectal cancer	1.37[b]	0.78, 2.39	NR
**Cardiovascular disease**									
Leermakers et al.	Lutein	Cohort & CC	Mixed	H vs L	6-13 y	Risk of stroke	0.82[a]	0.72, 0.93	NR*
Hajizadeh-Sharafabad et al.	Lutein & Zeaxanthin	RCT	Mixed	8-20 mg	8-32 wk	C-reactive protein (mg/L)	-0.3[c]	-0.45, -0.15	<0.001*
Hajizadeh-Sharafabad et al.	Lutein & Zeaxanthin	RCT	Mixed	8-27 mg	8-32 wk	IL-6 (pg/mL)	-0.40[c]	-1.36, 0.54	NR
**Mortality**									
Jayedi et al.	Alpha-carotene	Cohort	Serum	H vs L	2-26 y	Risk of all-cause mortality	0.68[a]	0.58, 0.78	NR*
Jayedi et al.	Alpha-carotene	Cohort	Serum	DR: Per 0.1 µmol/L	2-26 y	Risk of all-cause mortality	0.82[a]	0.75, 0.88	NR*
Jayedi et al.	Alpha-carotene	Cohort	Mixed	H vs L	2-26 y	Risk of all-cause mortality	0.79[a]	0.63, 0.94	NR*
Jayedi et al.	Lutein	Cohort	Mixed	H vs L	2-26 y	Risk of all-cause mortality	0.83[a]	0.66, 1.00	NR
Jayedi et al.	Lutein & Zeaxanthin	Cohort	Serum	H vs L	2-26 y	Risk of all-cause mortality	0.85[a]	0.74, 0.97	NR*
Jayedi et al.	Zeaxanthin	Cohort	Mixed	H vs L	2-26 y	Risk of all-cause mortality	0.94[a]	0.75, 1.13	NR
**Obesity & Type 2 diabetes**									
Jiang et al.	Alpha-carotene	Cohort	Diet	H vs L	10-23 y	Risk of Type 2 diabetes	0.91[a]	0.85, 0.96	NR*
Jiang et al.	Alpha-carotene	Cohort	Serum	DR: Per 0.2 µmol/L	8-10 y	Risk of Type 2 diabetes	0.65[a]	0.33, 1.26	NR
Jiang et al.	Alpha-carotene	Cohort	Serum	H vs L	8-10 y	Risk of Type 2 diabetes	0.71[a]	0.44, 1.16	NR

Jiang et al.	Alpha-carotene	Cohort	Diet	DR: Per 1 mg/d	10-23 y	Risk of Type 2 diabetes	0.88[a]	0.73, 1.06	NR
Jiang et al.	Lutein	Cohort	Serum	H vs L	8-10 y	Risk of Type 2 diabetes	0.65[a]	0.55, 0.77	NR*
Jiang et al.	Lutein	Cohort	Serum	DR: Per 0.2 µmol/L	8-10 y	Risk of Type 2 diabetes	0.79[a]	0.72, 0.86	NR*
Leermakers et al.	Lutein	Cohort & CC	Mixed	H vs L	10-23 y	Risk of Type 2 diabetes	0.97[a]	0.77, 1.22	NR
Jiang et al.	Zeaxanthin	Cohort	Serum	DR: Per 0.2 µmol/L	8-10 y	Risk of Type 2 diabetes	0.67[a]	0.41, 1.10	NR
Jiang et al.	Zeaxanthin	Cohort	Serum	H vs L	8-10 y	Risk of Type 2 diabetes	0.82[a]	0.63, 1.05	NR
**Bone health**									
Xu et al.	Alpha-carotene	Cohort	Mixed	H vs L	9-17 y	Risk of hip fracture	0.77[b]	0.55, 1.08	NR
Xu et al.	Lutein & Zeaxanthin	Cohort	Mixed	H vs L	9-17 y	Risk of hip fracture	0.94[b]	0.79, 1.11	NR
**Eye health**									
Jiang et al.	Alpha-carotene	Cohort	Diet	H vs L	5-15 y	Cataract incidence	0.96[a]	0.88, 1.05	0.37
Chong et al.	Alpha-carotene	Cohort	Mixed	H vs L	5-18 y	Incidence of early aged related macular degeneration	1.05[b]	0.87, 1.26	0.59
Cohen et al.	Alpha-carotene	CC	Mixed	H vs L	9 m	Preeclampsia	-0.01[d]	-0.16, 0.15	0.94
Jiang et al.	Lutein & Zeaxanthin	Cohort	Diet	H vs L	5-15 y	Cataract incidence	0.81[a]	0.75, 0.89	<0.001*
Chong et al.	Lutein & Zeaxanthin	Cohort	Mixed	H vs L	5-18 y	Incidence of early aged related macular degeneration	0.98[b]	0.86, 1.13	0.82
**Neurodegenerative disease**									
Takeda et al.	Alpha-carotene	Cohort & CC	Diet	H vs L	NR	Risk of Parkinson's disease	0.78[b]	0.54, 1.14	0.21
Takeda et al.	Lutein	Cohort & CC	Diet	H vs L	NR	Risk of Parkinson's disease	1.49[b]	0.83, 2.68	0.18
**Pregnancy health**									
Cohen et al.	Lutein	CC	Mixed	H vs L	9 m	Preeclampsia	-0.07[d]	-0.34, 0.20	0.61

CC = case control; DR = dose response; H vs L = highest dose (intervention) vs lowest dose (control); IL-6 = interleuken-6; NR = not reported; RCT = randomised controlled trial; wk = weeks; y = years

[a] Risk Ratio or relative risk; [b] Odds ratio; [c] Mean difference; [d] Standardized mean difference; [e] Hazard ratio.

* Significant difference favoring the intervention.

Table S6. Meta-analysis results of included SLRs for the health effects of pale yellow pigments found in fruit and vegetables.

AUTHOR	PIGMENT	DESIGN	TYPE	DOSE	DURATION	HEALTH OUTCOME	EFFECT SIZE	95% CI	P-VALUE
					**Cancer**				
Hui et al.	Flavonols	Cohort & CC	Diet	H vs L	1-8 y	Risk of breast cancer	0.88[a]	0.80, 0.96	NR*
Grosso et al.	Flavonols	Cohort	Diet	H vs L	8-13 y	Risk of breast cancer	0.96[a]	0.90, 1.03	NR
Grosso et al.	Flavonols	CC	Diet	H vs L	NR	Risk of breast cancer	0.81[a]	0.65, 1.02	NR
Woo & Kim a	Flavonols	Cohort & CC	Mixed	H vs L	NR	Risk of colorectal cancer	0.71[a]	0.63, 0.81	NR*
Cui et al.	Flavonols	Cohort & CC	Diet	H vs L	NR	Risk of esophageal cancer	0.89[b]	0.73, 1.09	0.28
Xie et al.	Flavonols	Cohort & CC	Diet	H vs L	2-27 y	Risk of gastric cancer	0.80[b]	0.70, 0.91	NR*
Grosso et al.	Flavonols	CC	Diet	H vs L	NR	Risk of liver cancer	1.18[a]	0.76, 1.83	NR
Grosso et al.	Flavonols	Cohort	Diet	H vs L	16-18 y	Risk of lung cancer	0.59[a]	0.18, 1.88	NR
Grosso et al.	Flavonols	Cohort	Diet	H vs L	8-16 y	Risk of pancreatic cancer	1.02[a]	0.86, 1.20	NR
Guo et al.	Flavonols	Cohort & CC	Diet	H vs L	7-17 y	Risk of prostate cancer	1.08[a]	1.00, 1.18	>0.05
Hua et al.	Flavonols	Cohort & CC	Diet	H vs L	NR	Risk of ovarian cancer	0.68[a]	0.58, 0.80	NR*
Woo & Kim b	Flavonols	Cohort & CC	Mixed	H vs L	NR	Risk of smoking related cancer	0.77[b]	0.63, 0.95	NR*
Grosso et al.	Kaempferol	CC	Diet	H vs L	NR	Risk of lung cancer	0.77[a]	0.62, 0.97	NR*
Grosso et al.	Myricetin	CC	Diet	H vs L	NR	Risk of lung cancer	0.83[a]	0.63, 1.10	NR
Grosso et al.	Quercitin	CC	Diet	H vs L	NR	Risk of lung cancer	0.73[a]	0.52, 1.02	NR
					**Cardiovascular disease**				
Fan et al.	Flavonols	Cohort	Diet	H vs L	4-21 y	Risk of CHD	0.88[a]	0.79, 0.98	NR*
Wang et al.	Flavonols	Cohort	Mixed	H vs L	5-28 y	Risk of CHD	0.91[a]	0.83, 1.01	NR
Wang et al.	Flavonols	Cohort	Mixed	DR: Per 20 mg/d	5-28 y	Risk of CHD	0.96[a]	0.90, 1.03	NR
Micek et al.	Flavonols	Cohort & CC	Diet	H vs L	4-23 y	Risk of CVD	0.85[a]	0.79, 0.91	NR*
Menezes et al.	Flavonols	RCT	Supplement	6-1000 mg	28-84 d	Diastolic blood pressure (mmHg)	-2.63[c]	-3.83, -1.42	<0.001*
Menezes et al.	Flavonols	RCT	Supplement	6-1000 mg	14-90 d	HDL cholesterol (mmol/L)	0.05[c]	0.02, 0.07	<0.001*

Godos et al.	Flavonols	Cohort	Diet	H vs L	4-14 y	Risk of hypertension	0.98[a]	0.91, 1.07	0.71
Menezes et al.	Flavonols	RCT	Supplement	6-1000 mg	14-90 d	LDL cholesterol (mmol/L)	-0.14[c]	-0.21, -0.07	<0.001*
Menezes et al.	Flavonols	RCT	Supplement	6-728 mg	28-84 d	Plasma glucose (mmol/L)	-0.18[c]	-0.29, -0.08	<0.001*
Wang et al.	Flavonols	Cohort	Mixed	DR: Per 20 mg/d	NR	Risk of stroke	0.86[a]	0.77, 0.96	NR*
Wang et al.	Flavonols	Cohort	Mixed	H vs L	NR	Risk of stroke	0.86[a]	0.75, 0.99	NR*
Menezes et al.	Flavonols	RCT	Supplement	6-1000 mg	28-84 d	Systolic blood pressure (mmHg)	-3.05[c]	-4.83, -1.27	0.001*
Menezes et al.	Flavonols	RCT	Supplement	6-1000 mg	14-90 d	Triacylglycerol's (mmol/L)	-0.11[c]	-0.18, -0.03	0.007*
Menezes et al.	Flavonols	RCT	Supplement	6-1000 mg	14-90 d	Total cholesterol (mmol/L)	-0.11[c]	-0.20, -0.02	0.021*
Mohammadi-Sartang et al.	Quercitin	RCT	Supplement	150-500 mg	6-10 wk	C-reactive protein (mg/L)	-0.33[c]	-0.50, -0.16	<0.001*
Ou et al.	Quercitin	RCT	Supplement	150-1000 mg	2-10 wk	C-reactive protein (mg/L)	-0.11[d]	-0.32, 0.10	0.31
Huang et al.	Quercitin	RCT	Supplement	30-1000 mg	2-12 wk	Diastolic blood pressure (mmHg)	-2.86[c]	-5.09, -0.63	0.01*
Guo et al.	Quercitin	RCT	Supplement	100-730 mg	4-12 wk	HDL cholesterol (mmol/L)	-0.06[d]	-0.19, 0.07	NR
Guo et al.	Quercitin	RCT	Supplement	100-730 mg	4-12 wk	LDL cholesterol (mmol/L)	-0.02[d]	-0.15, 0.11	NR
Huang et al.	Quercitin	RCT	Supplement	30-1000 mg	2-12 wk	HDL cholesterol (mmol/L)	0.02[c]	-0.02, 0.06	0.26
Ou et al.	Quercitin	RCT	Supplement	100-1000 mg	1-10 wk	IL-6 (pg/mL)	-0.58[d]	-1.24, 0.09	0.09
Peluso et al.	Quercitin	RCT	Supplement	489-1000 mg	14-84 d	IL-6 (pg/mL)	-0.064[c]	-0.24, 0.11	0.46
Huang et al.	Quercitin	RCT	Supplement	30-1000 mg	2-12 wk	LDL cholesterol (mmol/L)	0.02[c]	-0.07, 0.10	0.71
Huang et al.	Quercitin	RCT	Supplement	30-1000 mg	2-12 wk	Systolic blood pressure (mmHg)	-3.09[c]	-4.59, -1.59	<0.001*
Ou et al.	Quercitin	RCT	Supplement	150-500 mg	6-10 wk	TNF-alpha (pg/mL)	-0.25[d]	-0.62, 0.12	0.19
Peluso et al.	Quercitin	RCT	Supplement	150-1000 mg	14-84 d	TNF-alpha (pg/mL)	-0.032[c]	-0.20, 0.13	0.71
Guo et al.	Quercitin	RCT	Supplement	100-730 mg	4-12 wk	Total cholesterol (mmol/L)	0.04[d]	-0.09, 0.17	NR
Huang et al.	Quercitin	RCT	Supplement	30-1000 mg	2-12 wk	Total cholesterol (mmol/L)	0.00[c]	-0.09, 0.10	0.93
Guo et al.	Quercitin	RCT	Supplement	100-730 mg	4-12 wk	Triglycerides (mmol/L)	0.05[d]	-0.08, 0.18	NR
Huang et al.	Quercitin	RCT	Supplement	30-1000 mg	2-12 wk	Triglycerides (mmol/L)	-0.05[c]	-0.15, 0.05	0.25
**Mortality**									
Grosso et al.	Flavonols	Cohort	Mixed	H vs L	4-16 y	Risk of all-cause mortality	0.88[a]	0.69, 1.11	NR

Grosso et al.	Flavonols	Cohort	Mixed	DR: Per 10 mg/d	4-16 y	Risk of all-cause mortality	0.98[a]	0.95, 1.02	NR
Huxley et al.	Flavonols	Cohort	Mixed	H vs L	6-25 y	Risk of CHD mortality	0.80[a]	0.69, 0.93	NR*
Grosso et al.	Flavonols	Cohort	Mixed	H vs L	5-16 y	Risk of CVD mortality	0.79[a]	0.63, 0.99	NR*
Grosso et al.	Flavonols	Cohort	Mixed	DR: Per 10 mg/d	5-16 y	Risk of CVD mortality	0.87[a]	0.76, 0.99	NR*
**Obesity & Type 2 diabetes**									
Rienks et al.	Flavonols	Cohort	Diet	H vs L	4-24 y	Risk of Type 2 diabetes	0.92[e]	0.85, 0.98	NR*
Akhlaghi et al.	Flavonols	RCT	Supplement	150 mg	1-3 m	Waist circumference (cm)	-0.80[c]	-2.66, 1.06	0.40
Huang et al.	Quercitin	RCT	Supplement	100-1000 mg	1-3 m	Body mass index (kg/m2)	-0.04[c]	-0.54, 0.45	0.87
Huang et al.	Quercitin	RCT	Supplement	100-1000 mg	1-3 m	Body weight (kg)	-0.35[c]	-2.03, 1.33	0.68
Ostadmohammadi et al.	Quercitin	RCT	Supplement	100-1000 mg	4-12 wk	Fasting glucose (unit NR)	-0.53[c]	-1.81, 0.74	NR
Ostadmohammadi et al.	Quercitin	RCT	Supplement	162-250 mg	6-8 wk	HbA1c (unit NR)	0.06[c]	-0.01, 0.12	NR
Ostadmohammadi et al.	Quercitin	RCT	Supplement	162-1000 mg	6-12 wk	HOMA-IR (unit NR)	-0.24[c]	-0.49, 0.02	NR
Ostadmohammadi et al.	Quercitin	RCT	Supplement	100-1000 mg	6-12 wk	Insulin (unit NR)	-0.19[c]	-1.41, 1.04	NR
**Exercise**									
Pelletier et al.	Quercitin	RCT	Supplement	1000 mg	5-52 d	VO2 max (%)	1.94[c]	0.30, 3.59	0.02*
Somerville et al.	Quercitin	RCT	Supplement	500-1000 mg	7-56 d	Exercise performance (%)	2.82[c]	2.05, 3.58	<.001*

CC = case control; CVD = cardiovascular disease; CHD = coronary heart disease; d = days; DR = dose response; H vs L = highest dose (intervention) vs lowest dose (control); HbA1c = hemoglobin A1c; HOMA-IR = Homeostatic Model Assessment for Insulin Resistance; IL-6 = interleuken-6; m = months; NR = not reported; RCT = randomised controlled trial; TNF-alpha = tumor necrosis factor alpha; Total-C = total cholesterol; wk = weeks; y = years.

[a] Risk ratio; [b] Odds ratio; [c] Mean difference; [d] Standardized mean ratio; [e] Hazard Ratio

* Significant difference favoring the intervention

** Significant difference favoring control

Table S7. Meta-analysis results of included SLRs for the health effects of white pigments found in fruit and vegetables.

AUTHOR	PIGMENT	DESIGN	TYPE	DOSE	DURATION	HEALTH OUTCOME	EFFECT SIZE	95% CI	P-VALUE
					**Cancer**				
Grosso et al.	Flavones	Cohort	Diet	H vs L	9-13 y	Risk of breast cancer	1.01[b]	0.84, 1.21	NR
Grosso et al.	Flavones	CC	Diet	H vs L	NR	Risk of breast cancer	0.81[b]	0.68, 0.96	NR*
Hui et al.	Flavones	Cohort & CC	Diet	H vs L	1-3 y	Risk of breast cancer	0.83[b]	0.76, 0.91	NR*
Woo & Kim a	Flavones	Cohort & CC	Mixed	H vs L	NR	Risk of colorectal cancer	0.83[b]	0.63, 1.09	NR*
Grosso et al.	Flavones	CC	Diet	H vs L	NR	Risk of liver cancer	0.49[b]	0.30, 0.78	NR*
Grosso et al.	Flavones	Cohort	Diet	H vs L	16-18 y	Risk of lung cancer	0.94[b]	0.87, 1.02	NR
Grosso et al.	Flavones	Cohort	Diet	H vs L	11-16 y	Risk of pancreatic cancer	1.05[b]	0.94, 1.18	NR
Guo et al.	Flavones	Cohort & CC	Diet	H vs L	7-16 y	Risk of prostate cancer	1.04[b]	0.95, 1.14	>0.05
Cui et al.	Flavones	Cohort & CC	Diet	H vs L	NR	Risk of esophageal cancer	0.78[a]	0.64, 0.95	0.013*
Hua et al.	Flavones	Cohort & CC	Diet	H vs L	NR	Risk of ovarian cancer	0.86[b]	0.71, 1.03	NR
Woo & Kim b	Flavones	Cohort & CC	Mixed	H vs L	NR	Risk of smoking related cancer	0.77[a]	0.69, 0.85	NR*
					**Cardiovascular disease**				
Fan et al.	Flavones	Cohort	Diet	H vs L	4-21 y	Risk of CHD	0.94[b]	0.89, 0.99	NR*
Micek et al.	Flavones	Cohort & CC	Diet	H vs L	4-23 y	Risk of CVD	0.91[b]	0.82, 1.01	NR
Godos et al.	Flavones	Cohort	Diet	H vs L	4-14 y	Risk of hypertension	0.96[b]	0.90, 1.02	0.2
					**Mortality**				
Grosso et al.	Flavones	Cohort	Mixed	H vs L	4-16 y	Risk of all-cause mortality	0.86[b]	0.80, 0.93	NR*
Grosso et al.	Flavones	Cohort	Mixed	DR: Per 1 mg/d	4-16 y	Risk of all-cause mortality	0.96[b]	0.88, 1.04	NR
Grosso et al.	Flavones	Cohort	Mixed	H vs L	5-16 y	Risk of CVD mortality	0.85[b]	0.75, 0.96	NR*
Grosso et al.	Flavones	Cohort	Mixed	DR: Per 1 mg/d	5-16 y	Risk of CVD mortality	0.93[b]	0.90, 0.97	NR*
					**Obesity & Type 2 diabetes**				
Rienks et al.	Flavones	Cohort	Diet	H vs L	4-24 y	Risk of Type 2 diabetes	0.97[c]	0.87, 1.08	NR

CC = case control; CVD = cardiovascular disease; CHD = coronary heart disease; DR = dose response; H vs L = highest dose (intervention) vs lowest dose (control); IL-6 = interleuken-6; NR = not reported; RCT = randomised controlled trial; y = years.
[a] Odds ratio; [b] Risk ratio or relative risk; [c] Hazard ratio.
* Significant difference favoring the intervention.

Table S8. Meta-analysis results of included SLRs for the health effects of purple/blue pigments found in fruit and vegetables.

AUTHOR	PIGMENT	DESIGN	TYPE	DOSE	DURATION	HEALTH OUTCOME	EFFECT SIZE	95% CI	P-VALUE
					**Cancer**				
Grosso et al.	Anthocyanins	Cohort	Diet	H vs L	9-13 y	Risk of breast cancer	1.00[b]	0.91, 1.10	NR
Grosso et al.	Anthocyanins	CC	Diet	H vs L	NR	Risk of breast cancer	1.06[b]	0.96, 1.16	NR
Hui et al.	Anthocyanins	Cohort & CC	Diet	H vs L	1-3 y	Risk of breast cancer	0.97[b]	0.87, 1.08	NR
Yang et al.	Anthocyanins	Cohort	Diet	H vs L	1-12 y	Risk of gastric cancer	0.92[b]	0.81, 1.04	NR
Grosso et al.	Anthocyanins	CC	Diet	H vs L	NR	Risk of liver cancer	1.37[b]	0.89, 2.11	NR
Grosso et al.	Anthocyanins	Cohort	Diet	H vs L	16-18 y	Risk of lung cancer	0.97[b]	0.92, 1.02	NR
Cui et al.	Anthocyanins	Cohort & CC	Diet	H vs L	NR	Risk of esophageal cancer	0.60[a]	0.49, 0.74	<0.001*
Grosso et al.	Anthocyanins	Cohort	Diet	H vs L	11 y	Risk of pancreatic cancer	1.08[b]	0.96, 1.22	NR
Woo & Kim a	Anthocyanidins	Cohort & CC	Mixed	H vs L	NR	Risk of colorectal cancer	0.68[b]	0.56, 0.82	NR*
Guo et al.	Anthocyanidins	Cohort & CC	Diet	H vs L	7-16 y	Risk of prostate cancer	1.12[b]	1.03, 1.21	0.01**
Woo & Kim b	Anthocyanidins	Cohort	Mixed	H vs L	NR	Risk of smoking related cancer	0.89[a]	0.79, 1.01	NR
Grosso et al.	Proanthocyanidins	Cohort	Diet	H vs L	9-13 y	Risk of breast cancer	0.94[b]	0.87, 1.01	NR
Woo & Kim a	Proanthocyanidins	Cohort & CC	Mixed	H vs L	NR	Risk of colorectal cancer	0.72[b]	0.61, 0.85	NR*
Cui et al.	Proanthocyanidins	Cohort & CC	Diet	H vs L	NR	Risk of esophageal cancer	0.95[a]	0.72, 1.26	0.73
					**Cardiovascular disease**				
Fallah et al.	Anthocyanins	RCT	Supplement	11.2-1323 mg	6-24 wk	Adiponectin (µg/mL)	0.75[c]	0.23, 1.26	0.004*
Sangsefidi et al.	Anthocyanins	RCT	Mixed	0.77-531 mg	4-12 wk	Alanine Aminotransferase (U/L)	-1.08[c]	-3.96, 1.79	0.46
Shah & Shah	Anthocyanins	RCT	Mixed	80-320 mg	12-24 wk	Apolipoprotein A1 (mg/dL)	6.10[c]	4.51, 7.69	0.007*
Shah & Shah	Anthocyanins	RCT	Mixed	80-320 mg	12-24 wk	Apolipoprotein B (mg/dL)	-7.13[c]	-8.66, -5.59	0.006*
Sangsefidi et al.	Anthocyanins	RCT	Mixed	0.77-531 mg	4-12 wk	Aspartate Aminotransferase (U/L)	-1.71[c]	-3.60, 0.17	0.08
Daneshzad et al.	Anthocyanins	RCT	Supplement	31.45-600 mg	6-24 wk	Body mass index (kg/m2)	0.71[c]	-0.88, 2.30	0.38
Daneshzad et al.	Anthocyanins	RCT	Supplement	31.45-1050 mg	6-24 wk	Body weight (kg)	1.71[c]	-2.14, 5.55	0.39

Fan et al.	Anthocyanins	Cohort	Diet	H vs L	4-24 y	Risk of CHD	0.90[b]	0.83, 0.98	NR*
Kimble et al.	Anthocyanins	Cohort	Mixed	H vs L	6-26 y	Risk of CHD	0.91[b]	0.83, 0.99	NR*
Fallah et al.	Anthocyanins	RCT	Mixed	154-320 mg	12 wk	C-peptide (µg/L)	-0.02[c]	-0.20, 0.16	0.82
Sangsefidi et al.	Anthocyanins	RCT	Supplement	45.1-640 mg	3-24 wk	C-reactive protein (mg/L)	0.02[d]	-0.44, 0.48	0.94
Fallah et al.	Anthocyanins	RCT	Supplement	1.65-1323 mg	3-24 wk	C-reactive protein (mg/L)	-0.33[c]	-0.55, -0.11	0.003*
Shah & Shah	Anthocyanins	RCT	Mixed	80-320 mg	4 h - 12 wk	hs C-reactive protein (mg/dL)	0.16[c]	-0.06, 0.39	0.63
Micek et al.	Anthocyanins	Cohort & CC	Diet	H vs L	4-23 y	Risk of CVD	0.82[b]	0.70, 0.96	NR*
Daneshzad et al.	Anthocyanins	RCT	Supplement	31.45-500 mg	1-96 wk	Diastolic blood pressure (mmHg)	0.66[c]	-0.82, 2.14	0.38
Fallah et al.	Anthocyanins	RCT	Mixed	6.5-1024 mg	2 wk - 6 m	Fasting glucose (mg/dL)	-2.70[c]	-4.07, -1.33	<0.001*
Sangsefidi et al.	Anthocyanins	RCT	Mixed	67.5-531 mg	8-12 wk	Gamma-Glutamyl Transferase (U/L)	-3.75[c]	-11.63, 4.12	0.35
Fallah et al.	Anthocyanins	RCT	Mixed	7.35-742 mg	6-24 wk	HbA1c (%)	-0.21[c]	-0.39, -0.03	0.019*
Fallah et al.	Anthocyanins	RCT	Mixed	75.7-531 mg	4 wk - 6 m	HOMA-IR	-0.54[c]	-0.94, -0.14	0.008*
Araki et al.	Anthocyanins	RCT	Mixed	1.65-320 mg	4-24 wk	HDL cholesterol (mmol/L)	0.09[c]	0.02, 0.15	0.006*
Shah & Shah	Anthocyanins	RCT	Mixed	80-500 mg	4 h -24 wk	HDL cholesterol (mg/dL)	1.67[c]	0.80, 2.54	<0.001*
Godos et al.	Anthocyanins	Cohort	Diet	H vs L	4-14 y	Risk of hypertension	0.92[b]	0.88, 0.97	0.002*
Fallah et al.	Anthocyanins	RTC	Supplement	NR	NR	IL-1 beta (pg/mL)	1.71[c]	-3.77, 2.88	0.79
Fallah et al.	Anthocyanins	RCT	Supplement	6-1323 mg	3-12 wk	IL-6 (pg/mL)	-0.41[c]	-0.70, -0.13	0.004*
Shah & Shah	Anthocyanins	RCT	Mixed	200-500 mg	3-12 wk	IL-6 (pg/mL)	1.17[c]	0.80, 1.53	0.83
Fallah et al.	Anthocyanins	RCT	Supplement	1.6-640 mg	4-12 wk	Intracellular adhesion molecule-1 (ng/mL)	-52.4[c]	-85.7, -19.1	0.002*
Araki et al.	Anthocyanins	RCT	Mixed	1.65-320 mg	4-24 wk	LDL cholesterol (mmol/L)	-0.19[c]	-0.31, -0.06	0.003*
Fallah et al.	Anthocyanins	RCT	Mixed	154-320 mg	12-24 wk	Plasminogen activator inhibitor-1 (µg/L)	-5.09[c]	-9.45, -0.73	0.02*
Fallah et al.	Anthocyanins	RTC	Supplement	NR	NR	P-selectin (ng/mL)	-6.98[c]	-18.1, 4.15	0.22
Fallah et al.	Anthocyanins	RCT	Mixed	70.7-199.6 mg	4-12 wk	Resistin (µg/L)	-1.23[c]	-2.4, -0.05	0.04*
Kimble et al.	Anthocyanins	Cohort	Mixed	H vs L	5-41 y	Risk of stroke	1.00[b]	0.93, 1.07	NR
Fallah et al.	Anthocyanins	RCT	Supplement	6-742 mg	3-24 wk	TNF-alpha (pg/mL)	-0.64[c]	-1.18, -0.09	0.023*
Araki et al.	Anthocyanins	RCT	Mixed	1.65-320 mg	4-24 wk	Total cholesterol (mmol/L)	-0.19[c]	-0.42, 0.04	0.1

Araki et al.	Anthocyanins	RCT	Mixed	1.65-320 mg	4-24 wk	Triglycerides (mmol/L)	-0.20[c]	-0.33, -0.07	0.003*
Fallah et al.	Anthocyanins	RCT	Mixed	75.7-668 mg	4-24 wk	Serum insulin (mU/L)	0.33[c]	-0.18, 0.85	0.21
Daneshzad et al.	Anthocyanins	RCT	Supplement	31.45-500 mg	1-96 wk	Systolic blood pressure (mmHg)	1.80[c]	-0.92, 4.52	0.20
Fallah et al.	Anthocyanins	RCT	Supplement	1.6-640 mg	4-12 wk	Vascular adhesion molecule-1 (ng/mL)	-49.6[c]	-72.7, -26.5	<0.001*
Fairlie-Jones et al.	Anthocyanins	RCT	Mixed	1.34-581 mg	1 d - 6 m	Vascular reactivity	0.77[d]	0.37, 1.16	<0.001*
Fairlie-Jones et al.	Anthocyanins	RCT	Mixed	26-625 mg	1 wk - 4 m	Vascular stiffness	-0.19[d]	-0.39, 0.01	0.06
Daneshzad et al.	Anthocyanins	RCT	Supplement	31.45-320 mg	6-24 wk	Waist circumference (cm)	2.7[c]	-2.98, 8.83	0.35
Shah & Shah	Anthocyanins	RCT	Mixed	80-500 mg	4 hr -24 wk	LDL cholesterol (mg/dL)	-8.86[c]	-11.17, -20.02	<0.001*
Shah & Shah	Anthocyanins	RCT	Mixed	80-320 mg	12-24 wk	TNF-alpha (pg/mL)	-1.98[c]	-2.40, -1.55	0.001*
Shah & Shah	Anthocyanins	RCT	Mixed	80-500 mg	4 h -24 wk	Total cholesterol (mg/dL)	-3.55[c]	-10.8, 3.67	0.46
Shah & Shah	Anthocyanins	RCT	Mixed	80-320 mg	4 h -24 wk	Triglycerides (mg/dL)	-9.14[c]	-14.02, -4.13	0.014*
Ren et al.	Proanthocyanidins	RCT	Supplement	100-400 mg	5-16 wk	Diastolic blood pressure (mmHg)	-2.75[c]	-5.09, -0.41	0.021*
Ren et al.	Proanthocyanidins	RCT	Supplement	100-400 mg	5-16 wk	Mean arterial pressure (mmHg)	-3.37[c]	-6.72, -0.01	0.049*
Ren et al.	Proanthocyanidins	RCT	Supplement	100-400 mg	5-16 wk	Pulse pressure (mmHg)	-2.13[c]	-6.29, 2.03	0.32
Ren et al.	Proanthocyanidins	RCT	Supplement	100-400 mg	5-16 wk	Systolic blood pressure (mmHg)	-4.60[c]	-8.04, -1.16	0.009*
Ren et al.	Proanthocyanins	Cohort	Diet	H vs L	4-16 y	Risk of CHD	0.78[b]	0.65, 0.94	NR*
Micek et al.	Proanthocyanins	Cohort & CC	Diet	H vs L	4-16 y	Risk of CVD	0.83[b]	0.73, 0.95	NR*
**Mortality**									
Kimble et al.	Anthocyanins	Cohort	Mixed	H vs L	5-15 y	Risk of CVD mortality	0.92[b]	0.87, 0.97	NR*
Grosso et al.	Anthocyanidins	Cohort	Mixed	H vs L	4-16 y	Risk of all-cause mortality	0.89[b]	0.85, 0.94	NR*
Grosso et al.	Anthocyanidins	Cohort	Mixed	H vs L	5-16 y	Risk of CVD mortality	0.89[b]	0.83, 0.95	NR*
Grosso et al.	Anthocyanidins	Cohort	Mixed	DR: Per 10 mg/d	5-7 y	Risk of CVD mortality	0.94[b]	0.88, 0.99	NR*
Grosso et al.	Proanthocyanidins	Cohort	Mixed	H vs L	4-16 y	Risk of all-cause mortality	0.85[b]	0.70, 1.03	NR
Grosso et al.	Proanthocyanidins	Cohort	Mixed	H vs L	5-16 y	Risk of CVD mortality	0.89[b]	0.81, 0.97	NR*
Grosso et al.	Proanthocyanidins	Cohort	Mixed	DR: Per 10 mg/d	5-16 y	Risk of CVD mortality	0.97[b]	0.94, 1.00	NR
**Obesity & Type 2 diabetes**									

Park et al.	Anthocyanins	RCT	Supplement	28-500 mg	4-24 wk	Body mass index (kg/m2)	-0.36[c]	-0.58, -0.13	0.002*
Park et al.	Anthocyanins	RCT	Supplement	31-375 mg	4-8 wk	Body weight (kg)	0.15[c]	0.01, 0.29	0.04*
Rienks et al.	Anthocyanidins	Cohort	Diet	H vs L	4-20 y	Risk of Type 2 diabetes	0.86[e]	0.81, 0.91	NR*
Rienks et al.	Proanthocyanidins	Cohort	Diet	H vs L	NR	Risk of Type 2 diabetes	0.88[e]	0.75, 1.02	NR
					**Exercise**				
Bloedon et al.	Anthocyanins	RCT	Diet	NR	<2-6 wk	Catalase	0.17[d]	-0.51, 0.86	0.62
Kimble et al.	Anthocyanins	RCT	Diet	10-640 mg	3-28 d	Countermovement jump, 48hr post (ng/mL)	0.57[c]	0.04, 1.10	0.004*
Bloedon et al.	Anthocyanins	RCT	Diet	NR	<2-6 wk	C-reactive protein	-0.33[d]	-0.83, 0.17	0.19
Bloedon et al.	Anthocyanins	RCT	Diet	NR	<2-6 wk	hs C-reactive protein	-0.44[d]	-0.77, -0.11	0.009*
Kimble et al.	Anthocyanins	RCT	Mixed	8-547 mg	7-28 d	C-reactive protein, 48hr post	-0.24[d]	-0.57, 0.09	NR
Kimble et al.	Anthocyanins	RCT	Mixed	8-547 mg	7-30 d	Creatine kinase, 48hr post	-0.31[d]	-0.55, -0.08	<0.01*
Kimble et al.	Anthocyanins	RCT	Mixed	5-546 mg	3-30 d	Delayed onset muscle soreness, 48hr post	-0.20[d]	-0.40, 0.00	NR
Bloedon et al.	Anthocyanins	RCT	Diet	NR	<2-6 wk	Glutathione peroxidase	0.28[d]	-0.46, 1.01	0.46
Bloedon et al.	Anthocyanins	RCT	Diet	NR	<2-6 wk	Hiobarbituric acid species	-0.27[d]	-0.67, 0.13	0.19
Bloedon et al.	Anthocyanins	RCT	Diet	NR	<2-6 wk	IL-1b	0.081[d]	-0.31, 0.47	0.69
Bloedon et al.	Anthocyanins	RCT	Diet	NR	<2-6 wk	IL-6	-0.75[d]	-1.21, -0.29	0.002*
Kimble et al.	Anthocyanins	RCT	Mixed	8-640 mg	7-30 d	IL-6, 48hr post	-0.30[d]	-0.66, 0.06	NR
Bloedon et al.	Anthocyanins	RCT	Diet	NR	<2-6 wk	IL-8	-0.20[d]	-0.54, 0.14	0.25
Bloedon et al.	Anthocyanins	RCT	Diet	NR	<2-6 wk	Inflammation outcomes	-0.47[d]	-0.69, -0.25	<0.001*
Bloedon et al.	Anthocyanins	RCT	Diet	NR	<2-6 wk	Lipid hydroperoxides	-0.68[d]	-1.80, 0.44	0.23
Bloedon et al.	Anthocyanins	RCT	Diet	NR	<2-6 wk	Malondialdehyde	-0.65[d]	-1.02, -0.29	<.001*
Kimble et al.	Anthocyanins	RCT	Mixed	8-547 mg	3-30 d	Maximal voluntary contraction, 48hr post	0.67[d]	0.32, 1.02	NR*
Bloedon et al.	Anthocyanins	RCT	Diet	NR	<2-6 wk	Nitrotyrosine	-0.001[d]	-0.46, 0.46	1.00
Bloedon et al.	Anthocyanins	RCT	Diet	NR	<2-6 wk	Oxidative stress outcomes	-0.32[d]	-0.62, -0.02	0.04*
Bloedon et al.	Anthocyanins	RCT	Diet	NR	<2-6 wk	Protein carbonyls	-1.61[d]	-3.18, -0.40	0.05*
Bloedon et al.	Anthocyanins	RCT	Diet	NR	<2-6 wk	Superoxide dismutase	0.06[d]	-0.45, 0.56	0.83

Kimble et al.	Anthocyanins	RCT	Mixed	8-547 mg	8-28 d	Total antioxidant capacity, 48hr post	0.40[d]	-0.04, 0.84	NR
Bloedon et al.	Anthocyanins	RCT	Diet	NR	<2-6 wk	TNF-alpha	-0.45[d]	-0.82, -0.08	0.02*
Kimble et al.	Anthocyanins	RCT	Mixed	66-546 mg	7-10 d	TNF-alpha, 48hr post	-0.29[d]	-0.69, 0.11	NR
Bloedon et al.	Anthocyanins	RCT	Diet	NR	<2-6 wk	Total antioxidant capacity	0.49[d]	-0.09, 1.08	0.10
Bloedon et al.	Anthocyanins	RCT	Diet	NR	<2-6 wk	Uric acid	-0.12[d]	-0.93, 0.69	0.78

CC = case control; CVD = cardiovascular disease; CHD = coronary heart disease; d = days; DR = dose response; H vs L = highest dose (intervention) vs lowest dose (control); hs = high sensitivity; h = hours; HbA1c = hemoglobin A1c; HOMA-IR = Homeostatic Model Assessment for Insulin Resistance; IL-6 = interleuken-6; m = months; NR = not reported; RCT = randomised controlled trial; TNF-alpha = tumor necrosis factor alpha; Total-C = total cholesterol; wk = weeks; y = years.

[a] Odds ratio; [b] Risk ratio or relative risk; [c] Mean difference; [d] Standardized mean difference; [e] Hazard ratio.

* Significant difference favoring the intervention.

** Significant difference favoring control.

Table S9. Results of included RCTs and cohort studies for the health effects of green pigments found in fruit and vegetables.

AUTHOR	PIGMENT	DESIGN	TYPE	DOSE	DURATION	HEALTH OUTCOME	EFFECT SIZE	95% CI	P-VALUE
					**Cancer**				
Balder et al.	Chlorophyll	Cohort	Diet	H v L	9 y	Risk of colorectal cancer (M)	0.94[a]	0.72, 1.23	0.50
Balder et al.	Chlorophyll	Cohort	Diet	H v L	9 y	Risk of colorectal cancer (F)	1.20[a]	0.86, 1.69	0.24
Balder et al.	Chlorophyll	Cohort	Diet	H v L	9 y	Risk of colon cancer (M)	0.78[a]	0.57, 1.07	0.08
Balder et al.	Chlorophyll	Cohort	Diet	H v L	9 y	Risk of Colon cancer (F)	1.19[a]	0.80, 1.77	0.43
Balder et al.	Chlorophyll	Cohort	Diet	H v L	9 y	Risk of rectal cancer (M)	1.27[a]	0.80, 2.02	0.49
Balder et al.	Chlorophyll	Cohort	Diet	H v L	9 y	Risk of rectal cancer (F)	1.20[a]	0.68, 2.12	0.25
					**Seasonal Allergic Rhinitis**				
Fujiwara et al.	Chlorophyll	RCT	Supplement	0.7 mg	8 w	Rescue medication scores	-3.09[b]	-5.96, -0.22	0.045*
Fujiwara et al.	Chlorophyll	RCT	Supplement	0.7 mg	12 w	Rescue medication scores	-1.40[b]	NR	0.37
					**Cardiovascular disease**				
Montelius et al.	Chlorophyll	RCT	Supplement	3000 mg	12 w	Weight loss (kg)	-1.50[b]	-3.06, 0.06	0.06
Montelius et al.	Chlorophyll	RCT	Supplement	3000 mg	12 w	Fat free mass (kg)	-0.70[b]	-1.56, 0.16	0.11
Montelius et al.	Chlorophyll	RCT	Supplement	3000 mg	12 w	Body fat (kg)	-0.80[b]	-2.32, 0.72	0.29
Montelius et al.	Chlorophyll	RCT	Supplement	3000 mg	12 w	Body fat (%)	-0.20[b]	-1.56, 1.16	0.77
Montelius et al.	Chlorophyll	RCT	Supplement	3000 mg	12 w	Waist (cm)	-1.50[b]	-3.87, 0.87	0.21
Montelius et al.	Chlorophyll	RCT	Supplement	3000 mg	12 w	Insulin (mIE/L)	0.10[b]	-2.30, 2.50	0.93
Montelius et al.	Chlorophyll	RCT	Supplement	3000 mg	12 w	Glucose (mmol/L)	-0.16[b]	-0.42, 0.10	0.21
Montelius et al.	Chlorophyll	RCT	Supplement	3000 mg	12 w	Leptin (ng/mL)	-3.20[b]	-13.41, 7.01	0.53

F = females; H vs L = highest dose (intervention) vs lowest dose (control); M = males; NR = not reported; RCT = randomised controlled trial; wk = weeks; y = years

[a] Risk ratio or relative risk; [b] Mean difference.

* Significant difference favoring the intervention.

Table S10. PubMed systematic search strategy to identify umbrella reviews and systematic literature reviews of meta-analyses.

SEARCH PHASE	PIGMENT CLASS	INTERVENTION	STUDY DESIGN
1	Carotenoids	(caroten*[Title/Abstract] OR tetraterpene*[Title/Abstract] lycopene[Title/Abstract] OR cryptoxanthin[Title/Abstract] OR capsorubin[Title/Abstract] OR capsanthin[Title/Abstract] OR lutein[Title/Abstract] OR zeaxanthin[Title/Abstract] OR betaxanthin[Title/Abstract] OR violaxanthin[Title/Abstract] OR xanthophyll*[Title/Abstract] OR carotenoids[MeSH Terms] OR beta carotene[MeSH Terms] OR lycopene[MeSH Terms] OR xanthophylls[MeSH Terms]	AND (systematic[Title/Abstract] OR "meta-analysis"[Title/Abstract] OR "metaanalysis"[Title/Abstract] OR "meta-analyses"[Title/Abstract] OR "metaanalyses"[Title/Abstract] OR "literature review"[Title/Abstract] OR "meta-regression"[Title/Abstract] OR umbrella[Title/Abstract] OR systematic review[Publication Type] OR scientific integrity review[Publication Type] OR meta-analysis[Publication Type])
	Flavonoids	anthocyan*[Title/Abstract] OR cyanidin[Title/Abstract] OR malvidin[Title/Abstract] OR peonidin[Title/Abstract] OR delphinidin[Title/Abstract] OR pelargonidin[Title/Abstract] OR petunidin[Title/Abstract] OR aurone*[Title/Abstract] OR chalcone*[Title/Abstract] OR anthoxanthin*[Title/Abstract] OR flavonol*[Title/Abstract] OR kaempferol[Title/Abstract] OR quercetin[Title/Abstract] OR myricetin[Title/Abstract] OR flavone*[Title/Abstract] OR apigenin[Title/Abstract] OR luteolin[Title/Abstract] OR isoetin[Title/Abstract] OR tannin*[Title/Abstract] OR proanthocyanidin*[Title/Abstract] OR anthocyanins[MeSH Terms] OR flavonols[MeSH Terms] OR kaempferols[MeSH Terms] OR quercetin[MeSH Terms] OR chalcones[MeSH Terms] OR flavones[MeSH Terms] OR apigenin[MeSH Terms] OR luteolin[MeSH Terms] OR tannins[MeSH Terms] OR proanthocyanidins[MeSH Terms]	
	Betalains	betalain*[Title/Abstract] OR betacyanin*[Title/Abstract] OR betaxanthin*[Title/Abstract] OR betanin[Title/Abstract] OR indicaxanthin[Title/Abstract] OR vulgaxanthin[Title/Abstract] OR betalains[MeSH Terms] OR betacyanins[MeSH Terms] OR betaxanthins[MeSH Terms] OR	
	Chlorophylls	chlorophyll*[Title/Abstract] OR chlorophyll[MeSH Terms] OR chlorophyll A[MeSH Terms])	

Table S11. Systematic search strategy for EMBASE, CINAHL and Cochrane Library.[1]

DATABASE	SEARCH STRATEGY
EMBASE	(caroten*:ti,ab OR tetraterpene*:ti,ab OR lycopene:ti,ab OR cryptoxanthin:ti,ab OR capsorubin:ti,ab OR capsanthin :ti,ab OR lutein:ti,ab OR zeaxanthin:ti,ab OR betaxanthin:ti,ab OR violaxanthin:ti,ab OR xanthophyll*:ti,ab OR carotenoids/exp OR "betacarotene"/exp OR lycopene/exp OR xanthophylls/exp OR anthocyan*:ti,ab OR cyanidin:ti,ab OR malvidin:ti,ab OR peonidin:ti,ab OR delphinidin:ti,ab OR pelargonidin:ti,ab OR petunidin:ti,ab OR aurone*:ti,ab OR chalcone*:ti,ab OR anthoxanthin*:ti,ab OR flavonol*:ti,ab OR kaempferol:ti,ab OR quercetin:ti,ab OR myricetin: ti,ab OR flavone*:ti,ab OR apigenin:ti,ab OR luteolin:ti,ab OR isoetin:ti,ab OR tannin*:ti,ab OR proanthocyanidin*:ti, ,ab OR anthocyanins/exp OR flavonols/exp OR kaempferols/exp OR quercetin/exp OR chalcones/exp OR flavones/ exp OR apigenin/exp OR luteolin/exp OR tannins/exp OR proanthocyanidins/exp OR betalain*:ti,ab OR betacyanin *:ti,ab OR betaxanthin*:ti,ab OR betanin:ti,ab OR indicaxanthin:ti,ab OR vulgaxanthin:ti,ab OR betalains/exp OR betacyanins/exp OR betaxanthins/exp OR chlorophyll*:ti,ab OR chlorophyll/exp OR "chlorophyll A"/exp) AND (systematic:ti,ab OR meta-analysis:ti,ab OR metaanalysis:ti,ab OR meta- analyses:ti,ab OR metaanalyses:ti,ab OR "literature review":ti,ab OR meta-regression:ti,ab OR umbrella:ti,ab OR term:it OR term:it OR term:it)
CINAHL	((TI caroten* OR AB caroten*) OR (TI tetraterpene* OR AB tetraterpene*) OR (TI lycopene OR AB lycopene) OR (TI cryptoxanthin OR AB cryptoxanthin) OR (TI capsorubin OR AB capsorubin) OR (TI capsanthin OR AB capsanthin) OR (TI lutein OR AB lutein) OR (TI zeaxanthin OR AB zeaxanthin) OR (TI betaxanthin OR AB betaxanthin) OR (TI violaxanthin OR AB violaxanthin) OR (TI xanthophyll* OR AB xanthophyll*) OR (MH carotenoids+) OR (MH "beta carotene"+) OR (MH lycopene+) OR (MH xanthophylls+) OR (TI anthocyan* OR AB anthocyan*) OR (TI cyanidin OR AB cyanidin) OR (TI malvidin OR AB malvidin) OR (TI peonidin OR AB peonidin) OR (TI delphinidin OR AB delphinidin) OR (TI pelargonidin OR AB pelargonidin) OR (TI petunidin OR AB petunidin) OR (TI aurone* OR AB aurone*) OR (TI chalcone* OR AB chalcone*) OR (TI anthoxanthin* OR AB anthoxanthin*) OR (TI flavonol* OR AB flavonol*) OR (TI kaempferol OR AB kaempferol) OR (TI quercetin OR AB quercetin) OR (TI myricetin OR AB myricetin) OR (TI flavone* OR AB flavone*) OR (TI apigenin OR AB apigenin) OR (TI luteolin OR AB luteolin) OR (TI isoetin OR AB isoetin) OR (TI tannin* OR AB tannin*) OR (TI proanthocyanidin* OR AB proanthocyanidin*) OR (MH anthocyanins+) OR (TI betalain* OR AB betalain*) OR (TI betacyanin* OR AB betacyanin* OR (TI betaxanthin* OR AB betaxanthin*) OR (TI betanin OR AB betanin) OR (TI indicaxanthin OR AB indicaxanthin) OR (TI vulgaxanthin OR AB vulgaxanthin) OR (MH betalains+) OR (MH betacyanins+) OR (MH betaxanthins+) OR (TI chlorophyll* OR AB chlorophyll*) OR (MH chlorophyll+) OR (MH "chlorophyll A"+)) AND ((TI systematic OR AB systematic) OR (TI meta-analysis OR AB meta-mnalysis) OR (TI metaanalysis OR AB metaanalysis) OR (TI meta-analyses OR AB meta-analyses) OR (TI metaanalyses OR AB metaanalyses) OR (TI "literature review" OR AB "literature review") OR (TI meta-regression OR AB meta-regression) OR (TI umbrella OR AB umbrella) OR PT "systematic review" OR PT "scientific integrity review" OR PT meta-analysis)

The Cochrane Library	(caroten*:ti,ab OR tetraterpene*:ti,ab OR lycopene:ti,ab OR cryptoxanthin:ti,ab OR capsorubin:ti,ab OR capsanthin:ti,ab OR lutein:ti,ab OR zeaxanthin:ti,ab OR betaxanthin:ti,ab OR violaxanthin:ti,ab OR xanthophyll*:ti,ab OR [mh carotenoids] OR [mh “betacarotene”] OR [mh lycopene] OR [mh xanthophylls] OR anthocyan*:ti,ab OR cyanidin:ti,ab OR malvidin:ti,ab OR peonidin:ti,ab OR delphinidin:ti,ab OR pelargonidin:ti,ab OR petunidin:ti,ab OR aurone*:ti,ab OR chalcone*:ti,ab OR anthoxanthin*:ti,ab OR flavonol*:ti,ab OR kaempferol:ti,ab OR quercetin:ti,ab OR myricetin:ti,ab OR flavone*:ti,ab OR apigenin:ti,ab OR luteolin:ti,ab OR isoetin:ti,ab OR tannin*:ti,ab OR proanthocyanidin*:ti,ab OR [mh anthocyanins] OR [mh flavonols] OR [mh kaempferols] OR [mh quercetin] OR [mh chalcones] OR [mh flavones] OR [mh apigenin] OR [mh luteolin] OR [mh tannins] OR [mh proanthocyanidins] OR betalain*:ti,ab OR betacyanin*:ti,ab OR Betaxanthin*:ti,ab OR betanin:ti,ab OR indicaxanthin:ti,ab OR vulgaxanthin:ti,ab OR [mh betalains] OR [mh betacyanins] OR [mh betaxanthins] OR chlorophyll*:ti,ab OR [mh chlorophyll] OR [mh "chlorophyll A”]) AND (systematic:ti,ab OR meta-analysis:ti,ab OR metaanalysis:ti,ab OR meta-analyses:ti,ab OR metaanalyses:ti,ab OR "literature review":ti,ab OR meta-regression:ti,ab OR umbrella:ti,ab OR "systematic review":pt OR "scientific integrity review":pt OR meta-analysis:pt)

[1] Search strategy was designed in PubMed and translated to other databases using the Polygot Search Translator.

Table S12. Secondary systematic search strategy for Chlorophyll studies in PubMed, EMBASE, CINAHL and Cochrane Library.

DATABASE	SEARCH STRATEGY
PUBMED	((chlorophyll*[Title/Abstract]) OR (chlorophyll[MeSH Terms]) OR (chlorophyll A[MeSH Terms])) AND (("controlled trial"[Title/Abstract]) OR ("randomised trial"[Title/Abstract]) OR ("randomized trial"[Title/Abstract]) OR ("randomised study"[Title/Abstract]) OR ("randomized study"[Title/Abstract]) OR ("clinical trial"[Title/Abstract]) OR (RCT[Title/Abstract]) OR (placebo[Title/Abstract]) OR ("cohort study"[Title/Abstract]) OR (longitudinal[Title/Abstract]) OR ("prospective study"[Title/Abstract]) OR (clinical trial[Publication Type]) OR (controlled clinical trial[Publication Type]) OR (randomized clinical trial[Publication Type]) OR (observational study[Publication Type]) OR (comparative study[Publication Type]))
EMBASE	(chlorophyll*:ti,ab) AND (“controlled trial”:ti,ab OR “randomised trial”:ti,ab OR “randomized trial”:ti,ab OR “randomised study”:ti,ab OR “randomized study”:ti,ab OR "clinical trial”:ti,ab OR RCT:ti,ab OR “cohort study”:ti,ab OR longitudinal:ti,ab OR “prospective study”:ti,ab))
CINAHL	((AB chlorophyll* OR TI chlorophyll*) AND ((TI “controlled trial” OR AB “controlled trial”) OR (TI “randomised trial” OR AB “randomised trial”) OR (TI “randomized trial” OR AB “randomized trial”) OR (TI “randomised study” OR AB “randomised study”) OR (TI “randomized study” OR AB “randomized study”) OR (TI "clinical trial" OR AB “clinical trial”) OR (TI RCT OR AB RCT) OR (TI “cohort study” OR AB “cohort study”) OR (TI longitudinal OR AB longitudinal) OR (TI “prospective study” OR AB “prospective study))
The Cochrane Library	(chlorophyll*:ti,ab,kw AND ((controlled trial):ti,ab,kw OR (randomised trial):ti,ab,kw OR (randomized trial):ti,ab,kw OR (randomised study):ti,ab,kw OR (randomized study):ti,ab,kw OR (clinical trial):ti,ab,kw OR RCT:ti,ab,kw OR (cohort study):ti,ab,kw OR longitudinal:ti,ab,kw OR (prospective study):ti,ab,kw))

Table S13. Inter-rater reliability between reviewers at full text review.

REVIEWER A	REVIEWER B	A INCLUDE, B INCLUDE	A INCLUDE, B EXCLUDE	A EXCLUDE, B INCLUDE	A EXCLUDE, B EXCLUDE	PROPORTIONATE AGREEMENT	YES PROBABILITY	NO PROBABILITY	RANDOM AGREEMENT PROBABILITY	COHEN'S KAPPA
HM	MB	52	21	12	106	0.83	0.13	0.41	0.54	0.63
MB	SM	79	7	4	159	0.96	0.12	0.44	0.55	0.90
